# Driving forces in the assembly of lipid nanoparticles containing mRNA revealed by molecular dynamics simulations at acidic and physiological pH

**DOI:** 10.1038/s41598-025-20340-y

**Published:** 2025-10-21

**Authors:** Ari Hardianto, Regaputra Satria Janitra, Wahyu Widayat, Muhammad Yusuf, Neni Nurainy, Toto Subroto

**Affiliations:** 1 Department of Chemistry, Faculty of Mathematics and Natural Sciences, UniversitasPadjadjaran, Jatinangor, 45363 West Java Indonesia; 2 Research Center for Molecular Biotechnology and Bioinformatics, UniversitasPadjadjaran, Bandung, 40133 West Java Indonesia; 3 Faculty of Pharmacy, Mulawarman University, Samarinda, 75119 East Kalimantan Indonesia; 4 PT Bio Farma, Bandung, 40161 West Java Indonesia

**Keywords:** Lipid nanoparticle (LNP), Molecular dynamics (MD) simulation, LNP self-assembly, pH, Driving forces, Molecular self-assembly, Molecular dynamics

## Abstract

This study utilized all-atom molecular dynamics (MD) simulations to investigate the interactions and driving forces involved in the formation of mRNA-containing lipid nanoparticles (LNPs) at acidic pH (4.5) and physiological pH. Under the acidic condition, the LNP comprises mRNA, positively charged ionizable lipid (SM-102P), 1,2-distearoyl-sn-glycero-3-phosphocholine (DSPC), cholesterol, 1,2-dimyristoyl-rac-glycero-3-methoxypolyethylene glycol-2000 (DMG-PEG2000), and citrate ions with a charge of − 1. At physiological pH, it includes mRNA, both positively and neutrally charged ionizable lipids (SM-102P and SM-102N, respectively), DSPC, cholesterol, DMG-PEG2000, and citrate ions at − 1 and − 3 charges. MD analyses suggest that electrostatic forces play a significant role in mRNA and SM-102P interactions, which are crucial for mRNA encapsulation. Moreover, van der Waals forces are vital in the interactions between lipids during LNP formation, where at physiological pH, the lower polarity of SM-102N leads to stronger lipid interactions. Differences in the protonation states of ionizable lipids affect the hydrophobic interactions between lipid components in the LNP. Meanwhile, MD simulations in which all ionizable lipids are neutrally charged result in the mRNA not being encapsulated. Our finding offers insight into the self-assembly process of LNP, highlighting the crucial influence of pH and ionic strength on the encapsulation of mRNA by LNP.

**Supplementary Information:**

The online version contains supplementary material available at 10.1038/s41598-025-20340-y.

## Introduction

Lipid nanoparticles (LNPs) have emerged as a pivotal technology in drug delivery systems, particularly for nucleic acids like mRNA, due to their ability to protect these molecules from degradation and enhance intracellular delivery. LNPs are composed of ionizable lipids such as SM-102, helper lipids like 1,2-distearoyl-sn-glycero-3-phosphocholine (DSPC), cholesterol, and 1,2-dimyristoyl-rac-glycero-3-methoxypolyethylene glycol-2000 (DMG-PEG2000), which collectively facilitate the efficient delivery of mRNA into target cells^1^. The development of LNPs has significantly advanced the field of mRNA therapeutics, as evidenced by their critical role in the success of COVID-19 mRNA vaccines^2^. These vaccines utilize LNPs to encapsulate and deliver mRNA, ensuring stability and effective cellular uptake, which are crucial for the expression of the target protein and subsequent immune responses^3^. Moreover, LNPs have been employed in various therapeutic applications beyond vaccines, including cancer therapy, where they enable the targeted delivery of mRNA to specific cells or tissues, thereby improving therapeutic efficacy and reducing off-target effects^4,5^.

LNPs were synthesized by mixing nucleic acids with an acidic aqueous buffer, followed by the addition of lipid excipients dissolved in ethanol^6,7^. The initial formation of LNPs typically occurs in a low pH environment, such as a citrate buffer at pH 4.5, which stabilizes the positively charged state of ionizable lipids like SM-102, aiding in nanoparticle assembly. This acidic condition is pivotal as it allows the lipid components to form stable nanoparticles. Following their assembly, LNPs are transferred into a neutral pH buffer, such as phosphate-buffered saline (PBS) at physiological pH (7.4). The charge of SM-102 is altered by this buffer exchange, resulting in SM-102 with a neutral charge (SM-102N) at the outer LNP surface and a positively charged SM-102 (SM-102P) inside^8^. However, the detailed interaction between various lipid components during the assembly process in acidic and physiological pH is not fully understood^9^ including the roles of ionizable lipid, cholesterol, helper lipid, and DMG-PEG2000 in stabilizing the LNP structure.

Molecular dynamics (MD) simulations have been employed to investigate the self-assembly phenomena of lipids, proteins, and peptides^10^ and to study the influence of ionizable lipid types and lipid mixtures on the structure of LNPs in mRNA vaccines^11^. Fernandez-Luengo and colleagues utilized coarse-grained MD simulations to study the organization of lipid molecules within LNPs composed of tripalmitin lipid in water^12^. Additionally, the self-assembly mechanism of the nanoparticle-supported lipid bilayer (NPSLBL) has been explored using coarse-grained MD simulations^13^. Meanwhile, Hardianto and co-workers employed all-atom MD simulations to assess the impact of ethanol on the integrity of the mRNA vaccine LNP model, given its role as a solvent in the dissolution of lipid components during LNP production^7^.

In the present study, we employed all-atom MD simulations to gain detailed molecular insights into how different lipid components interact and the driving forces underlying the LNP assembly during mRNA encapsulation process at pH 4.5 and 7.4, in the presence of citrate ion species. At pH 4.5, the LNP system consists of mRNA, SM-102P, DSPC, cholesterol, and DMG-PEG2000, with citrate ions carrying a negative charge of − 1. At pH of 7.4, the LNP system includes mRNA, SM-102P (located in the interior), SM-102N (positioned on the exterior), DSPC, cholesterol, and DMG-PEG2000, with citrate ions carrying negative charges of − 1 and − 3. Understanding how these lipids contribute to the stability and functionality of LNPs could inform the design of more effective and robust LNP formulations.

## Materials and methods

### System preparation

To simulate the experimental preparation conditions of LNPs, MD simulations were performed under two different external environmental pH, mimicking the pH variation observed in wet lab setups^6^. Initially, the LNPs were generated in a citrate buffer solution at pH 4.5, a condition that maintains the positively charged state of SM-102P, facilitating the assembly of the lipid components into nanoparticles. This configuration is denoted as the Positive system. Subsequently, PBS was introduced to adjust the pH to 7.4, inducing a charge transition in SM-102: SM-102 molecules on the outer surface of LNPs become neutral (SM-102N), while those in the inner region of the LNP, with a pH of 4.5, remains positively charged (SM-102P). This second LNP configuration is denoted as the Neutral system. To investigate the effect of the SM-102 protonation state on mRNA encapsulation, we also created a third system in which all SM-102 molecules are in the neutral state (SM-102N), referred to as the All-Neutral system. Note that in the following sections of this paper, SM-102 will refer to the molecule regardless of its charge state.

The preparation of the simulation system was carried out using Packmol^14^ with a box dimension of 160 × 160 × 160 Å^3^. The number of molecules in the system was determined based on the ratio of protonated amine groups (N) of SM-102 at pH 4.5 to the number of negative charges from the phosphate groups (P) of the mRNA^7^. We used an N/P of 30:2 where one molecule of mRNA consisting of 21 nucleobases, namely 5’-UUCGUUGUCAAUGACGCUGCA-3’, which has a total negative charge of − 20 on the phosphate groups. The number of SM-102 molecules required to achieve an N/P ratio of 30:2 is 300. The ratio of SM-102:Cholesterol: DSPC: DMG-PEG2000 used was 50:38.5:10:1.5, which was found to be effective based on literature^6,15,16^. In this study, the LNP consists of 300 SM-102, 231 cholesterol, 60 DSPC, 9 DMG-PEG2000, and 1 mRNA. The number of SM-102P and SM-102N in the Neutral system is 62 and 238, respectively, obtained by adjusting the protonation state of SM-102P at a distance greater than 15 Å from mRNA. Table 1 presents the detailed composition of the simulation systems.

**Table 1:** Number of molecules LNP components in the LNP simulation systems. P and N denote SM-102P and SM-102N, respectively, while CHL is cholesterol. (− 1) and (− 3) represent citrate (− 1) and (− 3), respectively.

	SM-102		CHL	DSPC	DMG-PEG2000	mRNA	Citrate		Na^+^	Water
	P	N					(− 1)	(− 3)		
Positive system	300	–	231	60	9	1	300	0	20	97,924
Neutral system	62	238					37	187	556	97,464
All-Neutral system	–	300					0	300	920	97,024

### Molecular dynamics (MDs) simulation

MD simulations were performed using Amber 22^17^. The force field used for mRNA was OL3^18^. LIPID21^19^ was used for cholesterol and DSPC. For H_2_O and Na^+^, TIP3P^20^ was applied. SM-102, DMG-PEG2000, and citrate were parameterized using GAFF2 ^21^. We performed RESP charge parameterization for SM-102 and DMG-PEG 2000 at the ESP-HF/6-31G(d)//MP2/aug-cc-pVDZ level of theory, according to the structure fragments in Figure S1. Structure optimization was conducted using Gamess US VERSION 30 SEP 2020 (R2)^22^ while ESP charge calculations were done with Gaussian 09^23^. The parameters for citrate were obtained from our previous work^24^.

Minimization was performed in four stages with a restraint of 10 kcal mol^– 1^ Å^– 2^. The first and second stages of minimization used the steepest descent method for 10,000 cycles each. The first stage minimized mRNA, lipids, DMG-PEG2000, and ions, while the second stage was for H_2_O. The third and fourth stages of minimization used MD simulations in the NVE ensemble for 1 ns each. The third stage was for mRNA, lipids, DMG-PEG2000, and ions, whereas the fourth stage was for H_2_O.

System volume equilibration at 300 K was performed for 3 ns, with the last 2 ns of equilibration using the SHAKE algorithm for bonds involving hydrogen. System density equilibration was conducted for 10 ns with pressure regulation using the Berendsen barostat. During both equilibration stages, all system components except H_2_O and ions were restrained with a force constant of 0.1 kcal mol^–1^ Å².

Production simulation was conducted for 1000 ns. The simulation was performed in triplicate from the third minimization stage to the production stage. Each replicate was run with a different random seed, potentially leading to variations across trajectories of the same system. Non-bonded interaction calculations were performed using the PME method with a 10 Å cutoff. Temperature regulation was carried out using the Berendsen thermostat, while pressure regulation used the Monte Carlo barostat. The analysis of the total 9000 ns simulation trajectory was performed using CPPTRAJ^25^ particularly on the last 500-ns of MD trajectories unless otherwise specified. Interaction energy analyses were carried out through molecular mechanics with generalized Born and surface area (MMGBSA) calculations using MMPBSA.py^26^.

### Molecular visualization and graph generations

We utilized Biovia Discovery Studio Visualizer v21.10.020298 (Dassault Systèmes, San Diego, USA) and Visual Molecular Dynamics (VMD) 1.9.4^27^ for figure generation. The R packages of ggridge^28^ ggplot2^29^, gridExtra^30^ tidyr^31^ ggforce^32^ and ggpubr^33^ were used to make graphs on Jupyterlab 3.6.7 (Project Jupyter, Berkeley, CA, USA)^34^ under an R programming language environment version 4.3.1 (R Foundation for Statistical Computing, Vienna, Austria)^35^. Artworks were created using Inkscape 1.3 (0e150ed, 2023-07-21) (The Inkscape Project, Boston, MA, USA)^35^.

## Results and discussion

### Radius of gyration analysis

**Fig. 1:**
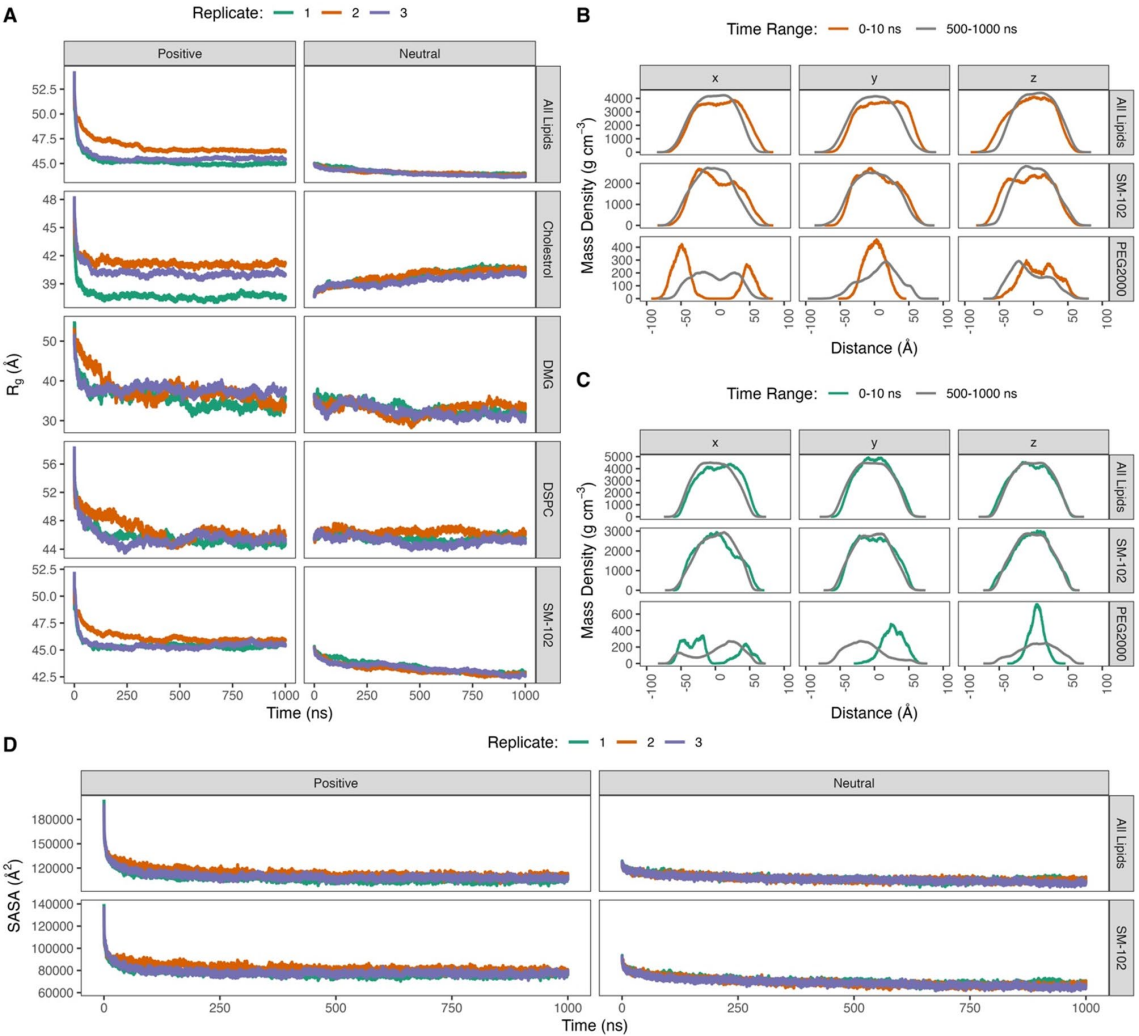
Gyration radius, mass density, and SASA profiles of two different LNP systems, Positive and Negative, from triplicate 1000-ns trajectories. (**A**) Gyration radius profiles for various lipid components. Time-based mass density profile comparison of ‘All Lipids’ (upper panel), SM-102 (middle panel), and PEG2000 (lower panel) in the (**B**) Positive and (**C**) Neutral systems in three axis directions. Mass density calculations were performed with the center of RNA as the origin. (**D**) SASA profiles of ‘All Lipids’ and SM-102 categories. The ‘All Lipids’ category covers the four lipid components including SM-102, DSPC, cholesterol, and DMG without PEG2000. DMG refers solely to 1,2-dimyristoyl-rac-glycero-3-methoxypolyethylene, excluding the PEG2000 moiety. The green, orange, and purple lines in (**A**) and (**D**) represent replicate 1, 2, and 3, respectively. The orange line in (**B**) denotes Positive system, while the green line in (**C**) is Neutral system. The orange and green lines represent the profiles at the time range of 0–10 ns in the Positive and Neutral systems, respectively, whereas the grey ones are those at last 500 ns.

In the first step, we analyzed the radius of gyration, computed as a scalar quantity representing the overall atomic distribution. To ensure reproducibility, three independent replicates were conducted for each system, yielding consistent results (Fig. 1A). Figure 1A illustrates the evolution of the radius of gyration for both individual lipid components and ‘All lipids’ (see the caption of Fig. 1) in two LNP systems: Positive and Neutral. In the Positive system, both individual lipid components and ‘All lipids’ display an initial rapid decrease in the radius of gyration (Fig. 1A), indicating a shift toward a more compact and ordered arrangement. This behavior suggests that, after the initial structural instability, the LNP components rearrange into a denser configuration. By contrast, the Neutral system exhibits a consistently tighter configuration for ‘All lipids’ throughout the simulation (44.09 ± 0.27 Å) compared to the Positive system (45.84 ± 0.93 Å), indicating reduced fluctuations and greater structural stability. Nonetheless, unlike other lipid components, cholesterol in the Neutral system exhibits an increased radius of gyration across all replicates, suggesting a potential structural perturbation that warrants further investigation to elucidate the underlying mechanisms.

An interesting aspect is that the gyration radius profiles for ‘All lipids’ closely mirror those of SM-102 in both LNP systems. This similarity suggests that the structural behavior of SM-102 plays a significant role in determining the overall compactness and stability of the LNP systems.

### Mass density analysis

Since the radius of gyration analysis indicates the more compact LNPs in both Positive and Neutral systems throughout the MD simulations, we proceeded with a mass density analysis of the ‘All Lipids’ and SM-102 lipid components over various time ranges (Figure S2, Figure S3, Figure S4, and Figure S5). Mass density profiles were calculated by combining trajectories from all three replicates for each system to enhance statistical robustness in resolving collective structural organization and improve clarity in visualization. As shown in Fig. 1B (upper panel), the ‘All Lipids’ in the Positive system during the late simulation period (grey line) generally exhibit shorter distances from the mRNA mass center compared to the early period (0–10 ns, orange line) across all axes, suggesting a decrease in LNP size. Interestingly, the SM-102 lipid component shows similar mass density profiles (Fig. 1B middle panel, Figure S4) to those of ‘All Lipids’, supporting our previous notion that SM-102 significantly influences the overall compactness and stability of the LNP. Additionally, the mass density on the x-axis of the PEG2000 moiety likely shifts toward the center of the LNP (Fig. 1B lower panel, grey line).

Mass density profiles of ‘All lipids’ in the Neutral system may experiences gradual decreases in distance from the LNP center of mass over time, particularly along the x and z axes (Figure S3). Consequently, the mass density profiles in the late time range (last 500 ns, grey line) show smaller distances compared to those in the early time range (0–10 ns, red line) (Fig. 1C upper panel, the x and z axes). The SM-102 lipid component also follows a similar trend of mass density decreases like those of ‘All Lipids’ (Fig. 1C middle panel, Figure S5), which further supports our previous notion that SM-102 significantly influences the overall compactness and stability of the LNP. Moreover, the mass density of PEG2000 is more distributed toward the LNP (Fig. 1C lower panel).

### Solvent accessibility surface area (SASA) analysis

The solvent accessible surface area (SASA) analysis may provide complementary insight into the compactness of LNPs. A lower SASA corresponds to reduced solvent accessibility and a more compact structure, whereas higher values reflect greater surface exposure^37^. In the Positive system, the SASA profiles for ‘All Lipids’ (Fig. 1D, left panel) decrease sharply within the first 10 ns across all replicates, demonstrating rapid reorganization into a tightly packed configuration. This compaction aligns with the gyration radius trends (Fig. 1A). Like those in gyration radius analysis, SM-102 (Fig. 1D, left panel) have a similar SASA profiles to ‘All Lipids’.

The ‘All Lipids’ in the Neutral system (Fig. 1D right panel) shows a gradual decrease of SASA values, indicating the LNP becoming more ordered, which agrees with our gyration radius analyses (Fig. 1A). SM-102 (Fig. 1D right panel), which combines positively (SM-102P) and neutrally charged SM-102 (SM-102N) molecules, similar SASA profiles to ‘All Lipids’. Further analysis on R03, the moiety of SM-102P bearing the positive charge, reveals increased SASA values in the first 10-ns, followed by fluctuated SASA values with a steady trend (Figure S6). Meanwhile, 03N, which is the R03 with a neutral charge in SM-102N, exhibits similar SASA profiles to SM-102 and ‘All Lipids’. Like the gyration radius analysis, the SASA of ‘All Lipids’ in the Neutral system (106,847.26 ± 3,953.77 Å^2^) indicates a more compact LNP structure compared to the Positive system (110,485.45 ± 6,396.65 Å^2^).

### Radial distribution function (RDF) analyses

**Fig. 2:**
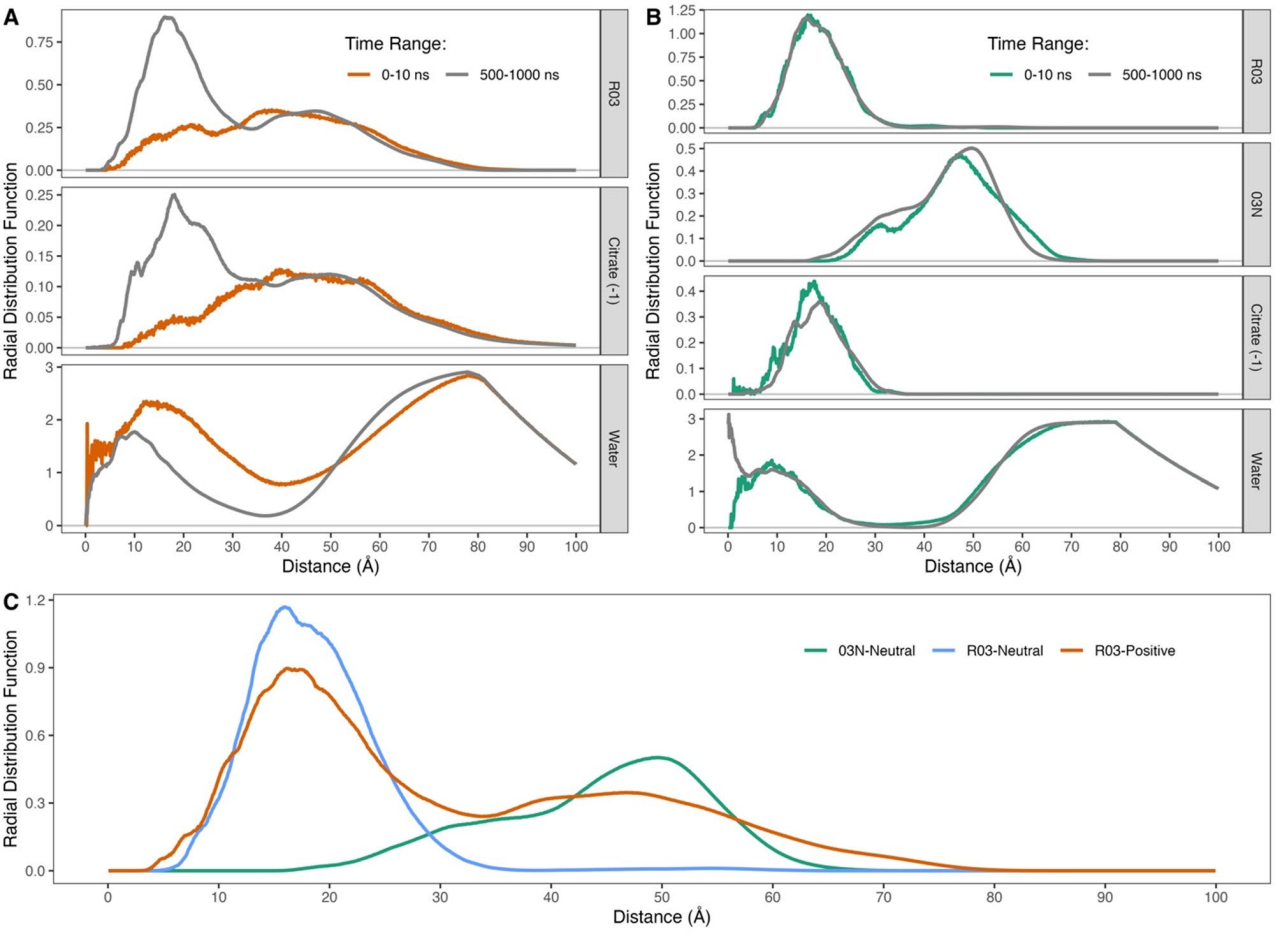
RDFs between center masses of mRNA and the moiety of SM-102 bearing charge, citrate (− 1), and water molecules in two different LNP systems from triplicate 1000-ns trajectories. (**A**) RDFs between mRNA and R03, citrate (− 1), and water molecules at time ranges of 0–10 ns and last 500 ns in the Positive system. (**B**) RDFs between mRNA and R03, 03N, citrate (− 1), and water molecules at time ranges of 0–10 ns and last 500 ns in the Neutral system. The orange line denotes Positive system, while the green line is Neutral system. The orange and green lines represent the profiles at the time range of 0–10 ns in the Positive and Neutral systems, respectively, whereas the grey ones are those at last 500 ns. R03 is the part of SM-102 bearing positive charge, whereas 03N is the part but in a neutral charge state. (**C**) RDFs between center masses of mRNA and R03 (blue) as well as 03N (green) in the Neutral system, and R03 (orange) in the Positive system at time ranges of last 500 ns. The illustration of the mRNA center of mass is shown in Figure S7.

We hypothesized that SM-102 molecules drive LNP compactness and stability via electrostatic interactions between the negatively charged mRNA and the protonated R03 headgroup of SM-102 at pH 4.5. Therefore, we calculated radial distribution functions (RDFs) between the center of mass of mRNA (Figure S7) and the R03 headgroup in the Positive system using combined trajectories from all replicates to enhance statistical resolution. As illustrated in Fig. 2A (upper panel), the RDFs of mRNA and R03 are distributed quite evenly between distances of 4.35 and 95.65 Å during the early time interval (0–10 ns). In the late time interval, the RDF is split into two: one at 2.85–33.75 Å and the other at 33.75–88.25 Å, hereafter denoted as the first and the second coordination shells, respectively. The RDF is more concentrated at the first coordination shell closer to the mRNA molecule. A more detailed analysis over various time ranges (Figure S8) shows an increasing RDF near the mRNA, indicating that SM-102 molecules approach the mRNA. This movement supports our notion that charge-charge interactions between mRNA and R03 affect LNP compactness. Furthermore, the lower distribution between 55.00 and 88.25 Å in the last 500 ns interval compared to the 0–10 ns interval suggests a decrease in the size of the LNP in the Positive system.

Additionally, we computed the RDF between the citrate ions, with a charge of − 1 (citrate (− 1)), and the center mass of mRNA (citrate (− 1)-mRNA) over various time intervals (Figure S9). Interestingly, the RDF profiles at the 0–10 ns and last 500 ns time intervals (Fig. 2A, middle panel) exhibit similar patterns to those of R03 and mRNA (Fig. 2A, upper panel). The RDF of citrate (− 1)-mRNA near the center mass of mRNA increases over time. RDF profiles over stepwise time ranges (Figure S9) also show patterns like those of R03 and mRNA. This observation emerges a notion that citrate (− 1) follows the movement of SM-102 due to electrostatic interaction. Interestingly, Fig. 2A also shows an overlap of the second coordination shell of R03-mRNA and citrate (− 1)-mRNA, indicating the presence of citrate (− 1) on the surface of the LNP. Citrate (− 1) ions may pair with SM-102 and potentially compensate for the repulsive forces between the positively charged SM-102 molecules, thereby increasing ionic strength. Moreover, we analyzed the number of LNP components in each coordination shell (Table 2) and calculated RDFs between the R03 headgroup (specifically its oxygen and nitrogen atoms, denoted as O^R03^ and N^R03^) and the oxygen atom of citrate (− 1) (O^citrate (−1)^) (Figure S10). The results confirm the presence of citrate (− 1) ions around R03 in the first and second coordination shells, with the highest RDF intensity at 2.65 and 2.85 Å for O^R03^-O^citrate (−1)^ and N^R03^-O^citrate (−1)^, respectively. Citrate (− 1) neutralizes the surface of the LNP, potentially playing a role in LNP aggregation and resulting in larger particle sizes. This notion is consistent with the findings of Nakamura and coworkers that the use of a citrate buffer during LNP formation resulted in relatively larger particles compared to the use of acetate and lactate buffers^38^.

**Table 2:** Composition of LNP constituents in each coordination shell for the positive and neutral systems.

	Positive System		Neutral System	
	1st Coordination Shell	2nd Coordination Shell	1st Coordination Shell	2nd Coordination Shell
R03	115.1 ± 9.3 (38.4%)	184.9 ± 9.3 (61.6%)	59.1 ± 1.2 (95.3%)	2.9 ± 1.2 (4.7%)
03N	-	-	126.7 ± 6.2 (53.2%)	111.3 ± 6.2 (46.8%)
R01 & R02	157.3 ± 17.3 (52.4%)	142.7 ± 17.3 (47.6%)	233.2 ± 6.4 (77.7%)	66.8 ± 6.4 (22.3%)
Cholesterol	111.9 ± 29.4 (48.4%)	119.1 ± 29.4 (51.6%)	174.5 ± 7.0 (75.6%)	56.5 ± 7.0 (24.4%)
DSPC	33.1 ± 11.1 (55.2%)	26.9 ± 11.1 (44.8%)	50.5 ± 3.9 (84.2%)	9.5 ± 3.9 (15.8%)
DMG	6.0 ± 1.5 (66.7%)	3.0 ± 1.5 (33.3%)	7.0 ± 0.8 (78.1%)	2.0 ± 0.8 (21.9%)
PEG 2000 (Monomers)	112.7 ± 40.6 (27.2%)	301.3 ± 40.6 (72.8%)	135.6 ± 22.6 (32.8%)	278.3 ± 22.6 (67.2%)
Na^+^	0.0 ± 0.1	12.8 ± 2.1	7.7 ± 4.6	267.6 ± 82.0
Citrate (− 1)	70.9 ± 8.1	217.0 ± 8.4	36.1 ± 0.8	0.6 ± 0.7
Citrate (− 3)	-	-	3.7 ± 1.9	100.4 ± 29.6
Water	1543.2 ± 693.8		1014.4 ± 129.0	

In the positive system, the first (2.85–33.75 Å) and second (33.75–88.25 Å) coordination shells are defined based on the RDF of R03-mRNA. In the neutral system, the first coordination shell (3.35–38.35 Å) is defined based on the RDF of R03-mRNA, while the second coordination shell (13.45–75.85 Å) is based on the RDF of 03N-mRNA. The coordination shells of 03N are determined from the RDF of 03N-mRNA and are divided into the first (13.45–38.35 Å) and second (38.35–75.85 Å) coordination shells.

Since the analyses of gyration radius, mass density, SASA, and RDF suggest that the LNP in the Positive system decreases in size over time, it is likely that water molecules inside the LNP are released. To further investigate this hypothesis, we calculated the RDF between water molecules and mRNA’s center of mass (water-mRNA) over different time ranges. The results in Fig. 2A (lower panel) and Figure S11 describe the distance between the center mass of mRNA and water molecules not atoms to atoms (see Figure S7). Figure 2A (lower panel) indicates that the RDF from 0.00 to 50.00 Å in the last 500 ns time range is lower compared to the 0–10 ns range. However, the distribution from 50.00 to 80.00 Å in the last 500 ns is higher than that in the 0–10 ns range, suggesting that most water molecules inside the LNP are released. This is confirmed by the fluctuation in the number of water molecules in the first coordination shell throughout the simulation (Figure S12). The high peak close to 0 Å in the last 500-ns range (Fig. 2A, lower panel) suggests that some water molecules form intensive interactions with the mRNA molecule. Furthermore, the RDF from 30 to 50 Å declines over time, reflecting that the channels or clefts in the LNP of the Positive system become smaller, which suggests the LNP becomes more compact.

**Fig. 3:**
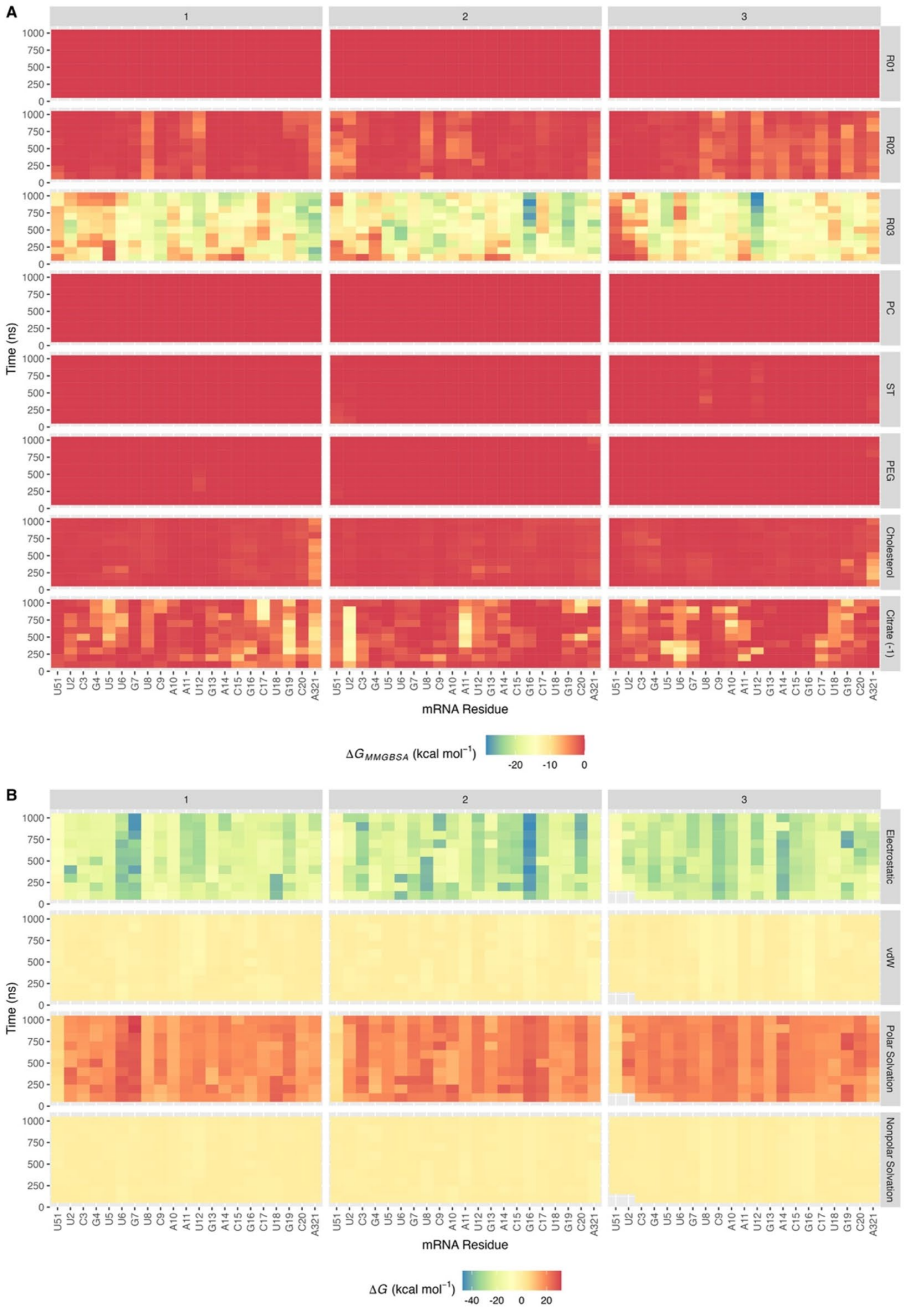
MMGBSA energy decomposition analysis (EDA) and interaction energy terms between mRNA and other components in the Positive system. (**A**) EDA between mRNA residues and other components. R01 and R02 are hydrophobic chains of SM-102 (see Figure S1), whereas R03 is the part of SM-102 bearing a positive charge; ST and PC refer to stearoyl (18:0) and phosphatidylcholine, respectively, which form DSPC; DMG is dimyristoyl (14:0) glycerol, while PEG is polyethylene glycol, which are parts of DMG-PEG2000 (see Figure S1); Citrate (− 1) is the citrate ion with a charge of − 1. (**B**) MMGBSA interaction energy terms between R03 and each mRNA residue. The energy values were computed at 100-ns intervals across 1000-ns trajectories. Numerical labels above each column in the graph denote replicates 1, 2, and 3.

For the LNP in the Neutral system, we initially organized the R03 headgroup at the inner region, while the neutral charged of R03 headgroup (03N) at the outer region (see Methods). Figure 2B (upper panel) shows that the RDF of R03 and the center of mass of mRNA (R03-mRNA) remains constant. Meanwhile, the RDF of 03N and the center of mass of mRNA (03N-mRNA) (Fig. 2B) slightly moves toward the mRNA in the last 500 ns compared to 0–10 ns. It does not have a sharp peak, which may be due to the migration of some 03N molecules toward the inner region of the LNP, where the distribution is classified into the coordination shells at 13.45–75.85 Å. Moreover, the RDF between 55.00 and 77.45 Å decreases at a time range of last 500 ns compared to that of 0–10 ns (Fig. 2B). Additionally, overlapping RDF profiles of R03-mRNA in both systems in the last 500 ns more clearly indicate that the exterior part of LNP in the Neutral system is contracted (Figs. 2C and 3N-Positive) compared to the Positive system (Fig. 2C, R03-Positive).

Like the profile in the LNP Positive system, the RDF profile of citrate (− 1)-mRNA in the Neutral system follows that of R03-mRNA (Fig. 2B, middle panel). Citrate (− 1) ions are around R03 as shown in Figure S13 and Table 2, with the highest RDF intensity at 2.65 and 2.75 Å for O^R03^-O^citrate (−1)^ and N^R03^-O^citrate (−1)^. Both RDFs of R03-mRNA and citrate (− 1)-mRNA slightly move away from the mRNA at the late time range. Meanwhile, the RDF of water-mRNA (Fig. 2B, lower panel; Figure S14) shows a decrease between 25 and 45 Å in the last 500 ns. This decline implies a possible reduction in the size of water permeation channels within the LNP, indicating increased structural compaction over time.

RDFs of citrate ions bearing charges of − 3 (citrate (− 3)) and the center mass of mRNA (citrate (− 3)-mRNA) show distributions of around 10.00 to 100.00 Å at the time range of 0–10 ns (Figure S14), suggesting that the diffusion of citrate (− 3) ions quickly occur at the early time range. Interestingly, at the time range of last 500 ns, the RDF is split to between around 5.85–36.35 Å and beyond 40.95 Å, leaving the distances between 36.45 and 40.85 Å having zero density. This observation suggests that some citrate (− 3) ions may move approaching SM-102P, with a positive charge, while others may move away from the outer surface of LNP. The analysis of the number of LNP components in each coordination shell (Table 2) shows the presence of a small number of citrate (− 3) ions in the first coordination shell. The majority of citrate (− 3) is located outside the LNP, as shown in Figure S15. Citrate (− 3) ions surrounding the outer surface coordinate with Na^+^ ions, which is confirmed by the RDF between Na^+^ and the oxygen atoms in citrate (− 3) (Na^+^-O^citrate (−3)^) (Figure S16), with the highest RDF intensity at 2.35 Å. Citrate ions are believed to act as a chelators for Na^+^ ions, and thus, using citrate as a buffering agent may potentially reduce mRNA hydrolysis^39^.

The analysis of the number of LNP components in each coordination shell (Table 2) in the Positive and Neutral systems show the presence of R03 around mRNA, supported by the RDF analysis between the R03 headgroup (N^R03^ and O^R03^) and the oxygen atoms of mRNA (O^mRNA^) (Figure S17). The highest RDF density values for N^R03^-O^mRNA^ and O^R03^-O^mRNA^ in the Positive and Neutral systems are 2.75 and 2.65 Å, respectively. In contrast, in the system where all R03 molecules are deprotonated to 03N (All-Neutral system), no sharp peak is observed in the RDF curve between the 03N headgroup (N^03N^ and O^03N^) and O^mRNA^. Additionally, its density profiles are lower than those in the Positive and Neutral systems, indicating a minimal presence of 03N around mRNA (Figure S17). This suggests the crucial role of electrostatic force in the interaction between mRNA and SM-102.

### Driving forces in LNP self-assembly

The MMGBSA energy decomposition analyses (EDAs) between mRNA and all components in the Positive system are visualized in Fig. 3A. The figure indicates that R03 exhibits the strongest binding energy with mRNA among others. Further analysis (Fig. 3B) shows that the strong binding energy arises from the electrostatic energy term, supporting our hypothesis that SM-102 influences LNP compactness and stability through interactions between negatively charged mRNA and positively charged R03. Positive values of polar solvation energy in Fig. 3B suggest that both R03 and mRNA are favorably solvated by water molecules, which may explain water molecules remaining in the interior of LNP (Fig. 2A, lower panel).

EDA (Fig. 3A, citrate (− 1)) also exhibits intriguing result that citrate (− 1) ions also display attractive binding energy with some mRNA residues, which is unexpected since their net charges are negative. Visual inspection (Figure S18A) unveils that citrate (− 1) ions interact with phosphate and base residues of the mRNA via hydrogen bonds (H-bonds). These results may explain the RDF peak of citrate (− 1)-mRNA between distances of 2.95 and 10.55 Å at last 500 ns (Fig. 2A, middle panel). Further visual analysis (Figure S18B) reveals citrate (− 1) ions bridging between the R03 headgroups via H-bonds and electrostatic interactions. Moreover, the analysis of MMGBSA interaction energy terms shows that some citrate (− 1) ions form highly electrostatic interactions with R03, as portrayed in Figure S19. These results support our previous hypothesis (in the Radial Distribution Analyses section above) that citrate (− 1) ions may pair with R03 and potentially compensate for the repulsive forces between the positively charged SM-102 molecules, thereby increasing ionic strength. These results also address our other hypothesis, from RDF analyses, that citrate (− 1) ions follow the movement of SM-102 due to electrostatic interaction.

**Fig. 4:**
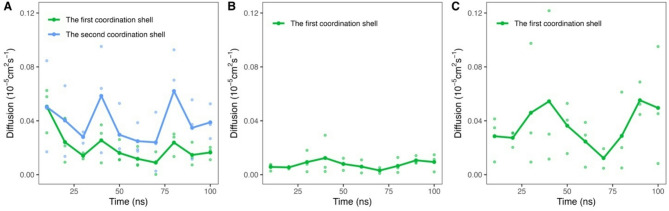
Diffusion constants of SM-102 in the Positive and Neutral systems, where R03 and 03N act as the probes, at 10-ns intervals over the first 100 ns. (**A**) The diffusion constants of R03 in the Positive system. (**B**) The diffusion constants of R03 in Neutral system. (**C**) The diffusion constants of 03N in the Neutral system. Diffusion constants were calculated from triplicate trajectories. The plotted lines represent average diffusion constant values, while individual data points show results from each replicate trajectory. Color-coding distinguishes coordination shells: green for the first coordination shell and blue for the second.

**Fig. 5:**
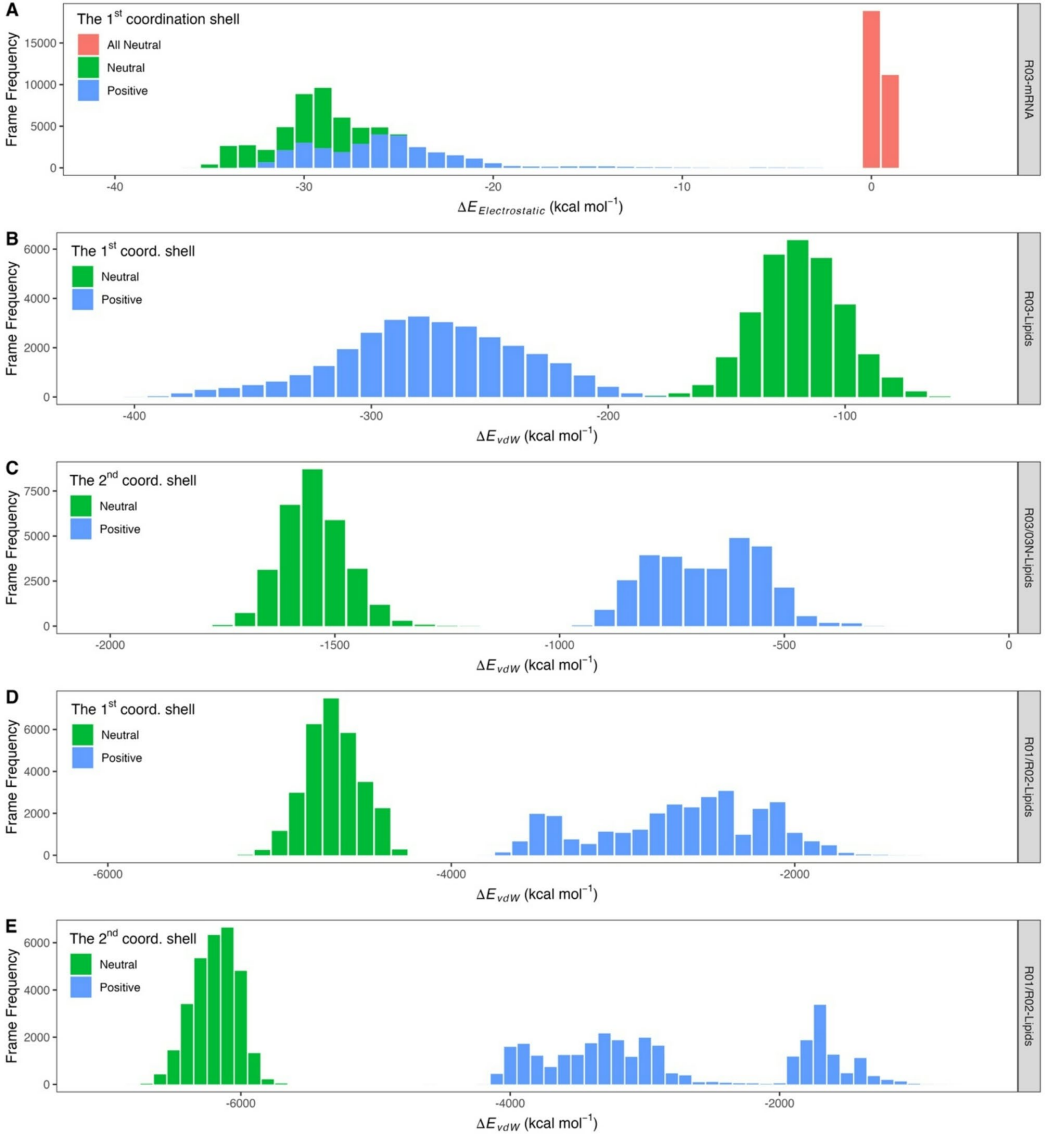
Linear Interaction Energy (LIE) histograms for the Positive, Neutral, and All‑Neutral systems. (**A**) Electrostatic interaction energy between residue R03 and mRNA within the first coordination shell of the Positive, Neutral, and All‑Neutral systems. (**B**) Van der Waals (vdW) interaction energy between R03 and surrounding lipid components in the first coordination shell of the Positive and Neutral systems. (**C**) vdW interaction energy between (i) R03 and other lipid components in the second coordination shell of the Positive system, and (ii) 03N and other lipid components in the second coordination shell of the Neutral system. (**D**) vdW interaction energy between the hydrophobic portions of SM‑102 (residues R01 and R02; see Figure S1) and lipid components in the first coordination shell of the Positive system. (**E**) vdW interaction energy between the hydrophobic portions of SM‑102 and lipid components in the second coordination shell of the Positive system. The Positive, Neutral, and All‑Neutral systems are represented in blue, green, and red, respectively.

To gain a deeper understanding of the LNP self-assembly process, we calculated the diffusion constants (Fig. 4A, B, C, and Figure S20) and the linear interaction energy (LIE) (Fig. 5A, B, C, D, and E). R03 exhibits the highest diffusion constant during the initial 10 ns simulation period within the first coordination shell (Fig. 4A, green), corresponding to the movement of R03 towards mRNA in the Positive system. R03 and mRNA in the first coordination shell experience an electrostatic attractive force, as shown in the LIE plot (Fig. 5A, blue histogram), while those in the second coordination shell do not (data not shown). R03 also attracts some DSPC molecules, through phosphatidylcholine moieties via electrostatic interactions (Figure S21). From LIE (Fig. 5B and C, blue histogram) and EDA (Figure S22), we also found that SM-102P and other lipids in the Positive system attract each other through van der Waals (vdW) forces. In the LNP formation, vdW forces between lipids may play a crucial role in LNP self-assembly, which is consistent with the work reported by Maeki and coworkers^40^. The self-assembly of lipid hydrophobic chains is a critical stage in LNP formation, driven by increased solvent polarity, due to ethanol dilution by an aqueous buffer, to reduce surface energy^40,41^.

In the Neutral system, the profiles of EDA and MMGBSA interaction energy terms between mRNA residues and all LNP components (Figure S23A and B) follows a similar pattern to those in the Positive system (Fig. 3A and B). Like in the Positive system, electrostatic interactions significantly contribute to the total interaction energy between R03 and mRNA. Meanwhile, in diffusion constant, R03 exhibits low fluctuations (Fig. 4B). The high fluctuations in the diffusion constant of 03N during the first 100 ns of the simulation (Fig. 4C) are related to LNP contraction. This contraction is the result of van der Waals (vdW) strengthening among lipids due to the protonation change of R03 to its neutral form (03N) as the pH shifts to 7.4. In the Neutral system, vdW interactions of SM-102 and other lipid components in the second coordination shell are stronger than those in the Positive system (Fig. 5C, D, and E). Charge alteration to neutral causes 03N more hydrophobic than R03, as reflected by their dipole moments of 1.1763 and 6.8580 Debye, respectively, leading to a greater tendency for SM-102N to interact with other lipids to reduce surface energy. This enhanced lipid–lipid affinity may also explain the observed increase in the radius of gyration of cholesterol in the Neutral system (Fig. 1A). Notably, ethanol, which is commonly used in LNP production, can disrupt hydrophobic interactions^42^ thus potentially affecting LNP formation. Experiments confirmed that rapid ethanol dilution is crucial for producing small and uniform LNP sizes^40,43^.

**Fig. 6:**
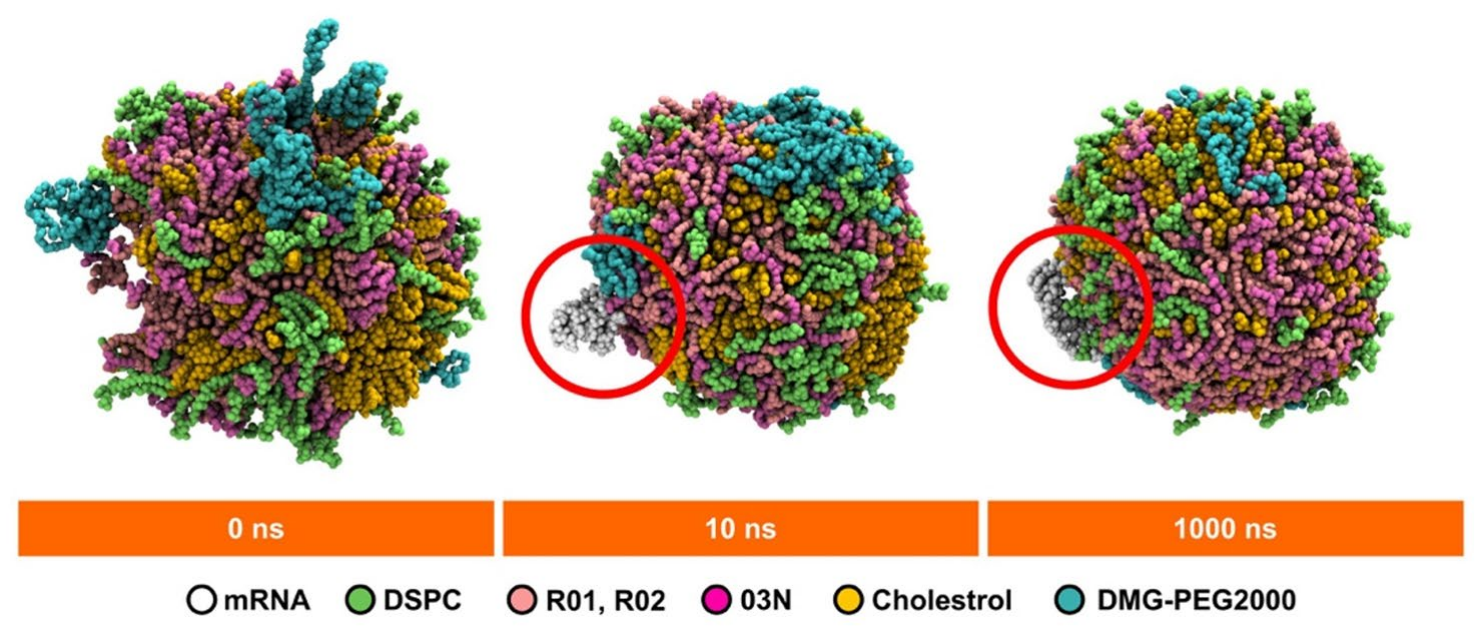
Representative visualization of the mRNA-LNP system with all ionizable SM-102 lipids in a neutral charged state.

Our MD simulations primarily highlight the critical role of the protonation state of ionizable lipids in mRNA encapsulation and LNP formation. LIE calculations for the All-Neutral system, in which all R03 moiety are deprotonated to 03N, indicate the absence of electrostatic interactions between 03N and mRNA (Fig. 5A, red). This finding is consistent with RDF calculations between N^03N^, O^03N^, and O^mRNA^ (Figure S17). Consequently, in the absence of positively charged lipids, the mRNA remains unencapsulated (Fig. 6).

Ionic strength, pH, and solvent polarity are interrelated parameters that may modulate mRNA payload and LNP size. Elevated ionic strength is known to shift the protonation equilibrium of ionizable lipids toward their neutral form^45^ and to decrease encapsulation efficiency in citrate‑based formulations^46,47^. We hypothesize that higher pH and ionic strength in experimental formulations would increase the fraction of neutral SM‑102. Additionally, higher ionic strength may promote stronger interactions between mRNA and counterions such as Na^+^, reducing the efficiency of encapsulation and promoting aggregation^38,44,48^. Meanwhile, adjusting the solvent polarity is crucial to determine the ratio of water to ethanol in LNP formation, which is suspected to be related to the proportion of SM-102P and SM-102N. Future simulations exploring variations in ionic strength, pH, and solvent polarity will be necessary to dissect their relative contributions to mRNA encapsulation.

### Visual analysis

**Fig. 7:**
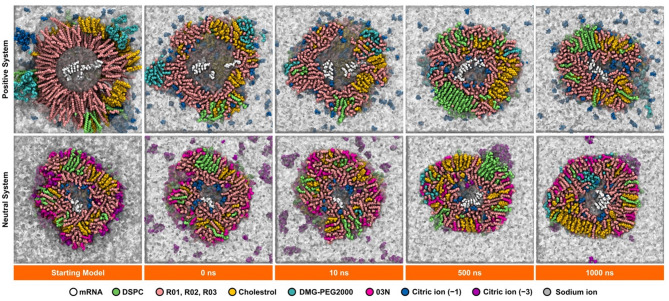
Representative visualizations of LNPs in Positive (top) and Neutral (bottom) systems. mRNA is in white vdW representation; DSPC is in green vdW representation; R01, R02, and R03 in pink vdW representation; Cholesterol is in yellow vdW representation; DMG-PEG2000 is in cyan vdW representation; Citric (− 1) ion is blue vdW representation; Citric (− 3) ion is vdW representation; Sodium ion is in grey vdW representation; Water is in white transparent surface representation. Starting models were generated by using Packmol. The time stamps indicate the time frames of MD trajectory production stages.

In the Positive system, the LNP undergoes dramatic changes throughout the simulation (Fig. 7, upper panel). The initial configuration reveals water molecules within the lipid nanoparticle (LNP). During the first 10 ns of the simulation, these water molecules migrate out of the LNP through a large channel that formed, providing a pathway for their escape. This migration of water molecules may be due to strong electrostatic interactions between R03 and mRNA (Fig. 3B), squeezing the LNP assembly. As the simulation progressed, the channel is gradually closed, trapping some water molecules inside. This closing coincides with increases electrostatics interactions between R03 and mRNA (Fig. 3B) as well as the hydrophobic parts of SM-102 (R01 and R02) and other lipids (Figure S24). These interactions suggest that the reorganization within the LNP, driven by these forces, stabilized the structure and limited further water egress. The positive values of polar solvation energy suggest favorable solvation by water molecules, which may explain the retention of some water molecules in the interior of the LNP (Fig. 2A, lower panel, and Fig. 7). Additionally, some citrate (− 1) ions are adsorbed onto the surface of the LNP, likely due to pairing with R03 molecules residing on the LNP exterior (Figure S18B). Furthermore, as the simulation progresses, DMG-PEG2000 becomes increasingly buried within the LNP, while the PEG2000 segment folds (Fig. 7, upper panel, cyan), aligning with other studies that report PEG2000 adopting a prolate spheroidal^49^.

In the Neutral system, MD production trajectory visualizations differ from those in the Positive system (Fig. 7). This difference may be attributed to the neutralization of R03 to 03N on the LNP exterior, which leads to stronger vdW interactions among lipids and enhances the integration of SM-102N with other lipids. Moreover, conversion of R03 to 03N causes citrate (− 3) ions desorbing from the LNP surface (Figure S15) and forming clusters with sodium ions as discussed in the Radial Distribution Analysis section.

## Conclusion

This study provides a comprehensive understanding of the molecular driving forces involved in the assembly of mRNA-containing LNP at acidic (pH 4.5) and physiological (pH 7.4) conditions through all-atom MD simulations. The results highlight the significant role of electrostatic interactions between the positively charged part of SM-102 (R03) and mRNA in mRNA encapsulation at acidic pH. These electrostatic interactions also contribute to the compactness and overall integrity of the mRNA-containing-LNP, likely initiating LNP formation by attracting SM-102P molecules to mRNA, while the hydrophobic chains of SM-102P recruit other lipids through vdW interactions. The enhancement of vdW interactions among lipid components, particularly in the Neutral system where SM-102 is neutralized (03N), further emphasizes the influence of charge states on the structural behavior and lipid integration within LNPs.

The simulations revealed that the conversion of R03 to its neutral form (03N) in the Neutral system not only strengthens vdW interactions but also leads to the desorption of citrate (− 3) ions from the LNP surface. This change contributes to the slight expansion of the LNP’s interior, while the exterior becomes more compact, enhancing LNP stability at physiological pH. Additionally, the study underscores the importance of solvent conditions, such as ethanol dilution, in influencing LNP formation and stability.

Taken together, these results offer important understanding of molecular mechanisms of LNP self-assembly and stability, providing guidelines for designing and optimizing LNP formulations for mRNA delivery. Our simulations show that keeping a ratio of positively charged SM-102 (SM-102P) in the early stage of assembly is essential for successful mRNA encapsulation owing to strong electrostatic interactions, whereas a switch to neutral SM-102 (SM-102N) facilitates lipid–lipid interaction and stabilizes the nanoparticle at physiological pH. These observations imply that adjustment of the protonation state of ionizable lipids through pH control and ethanol dilution rate can be an important strategy to achieve maximal mRNA loading and structural stability of LNPs.

## Supplementary Information

Below is the link to the electronic supplementary material.

Supplementary Material 1

## Data Availability

The simulation data can be requested to AH via a.hardianto@unpad.ac.id.
