# Supplementary material for: Driving forces in the assembly of lipid nanoparticles containing mRNA revealed by molecular dynamics simulations at acidic and physiological pH
Source: Sci Rep. 2025 Oct 21;15:36645. doi: 10.1038/s41598-025-20340-y (PMC12541038; doi:10.1038/s41598-025-20340-y)
Supplement: Supplementary file 1 — Supplementary Material 1 [file 41598_2025_20340_MOESM1_ESM.docx]

**Supporting Information**

**Driving Forces in the Assembly of Lipid Nanoparticles Containing mRNA Revealed by Molecular Dynamics Simulations at Acidic and Physiological pH**

**Ari Hardianto,^*a,b^ Regaputra Satria Janitra,^b^ Wahyu Widayat,^b,c^ Muhammad Yusuf,^a,b^ Neni Nurainy,^d^ and Toto Subroto^‡a,b^**

*^a^Department of Chemistry, Faculty of Mathematics and Natural Sciences, Universitas Padjadjaran, Jatinangor 45363, West Java, Indonesia.*

*^b^Research Center for Molecular Biotechnology and Bioinformatics, Universitas Padjadjaran, Bandung 40133, West Java, Indonesia.*

*^c^Faculty of Pharmacy, Mulawarman University, Samarinda 75119, East Kalimantan, Indonesia.*

*^d^PT Bio Farma, West Java 40161, Indonesia.*

*a.hardianto@unpad.ac.id

**^‡^**t.subroto@unpad.ac.id


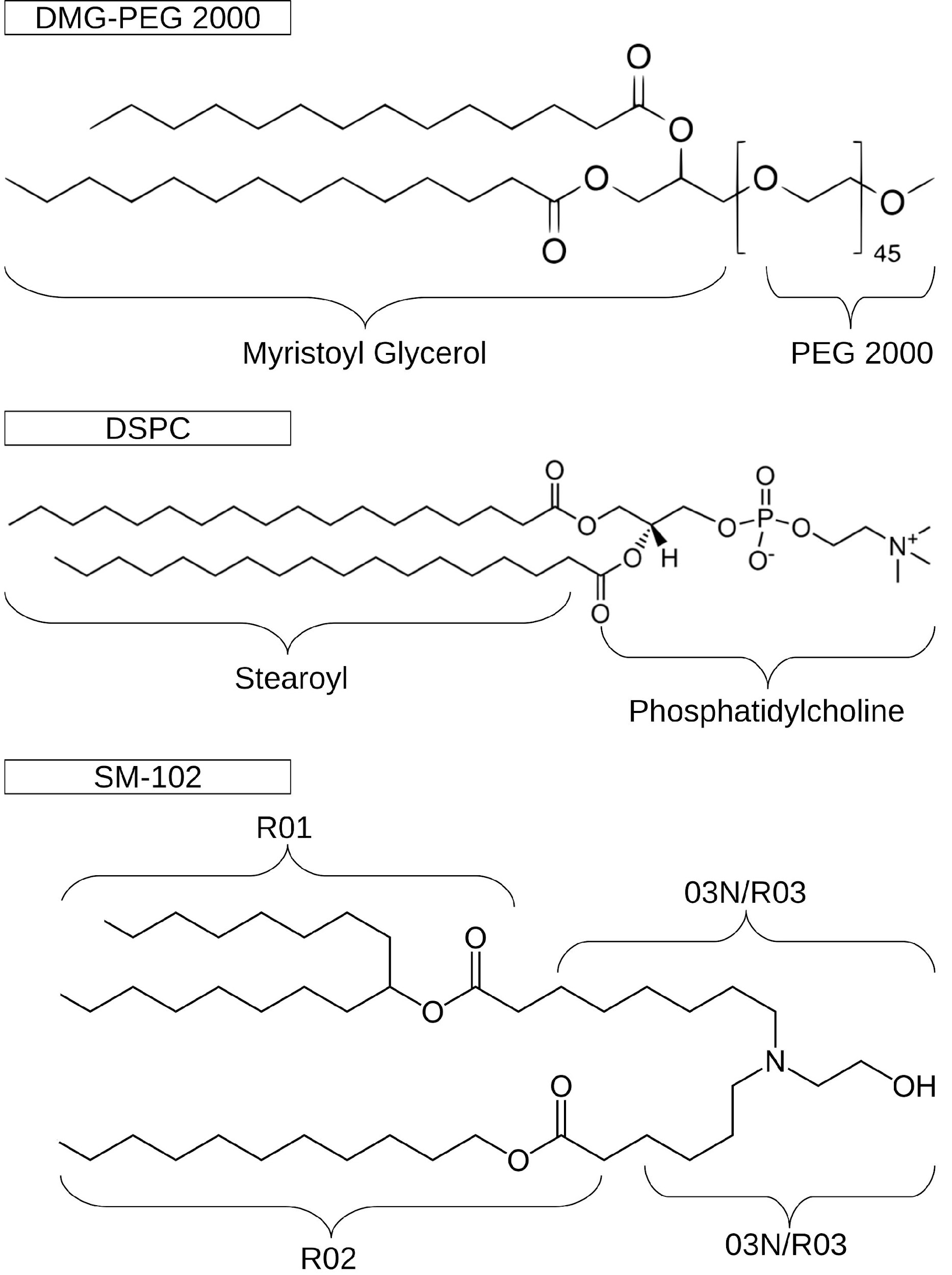


**Figure S1** The chemical structures of DMG-PEG2000, DSPC, and SM-102. The designation of 03N and R03 in SM-102 depends on the protonation state of the N atom, R03 is the protonated form of 03N.


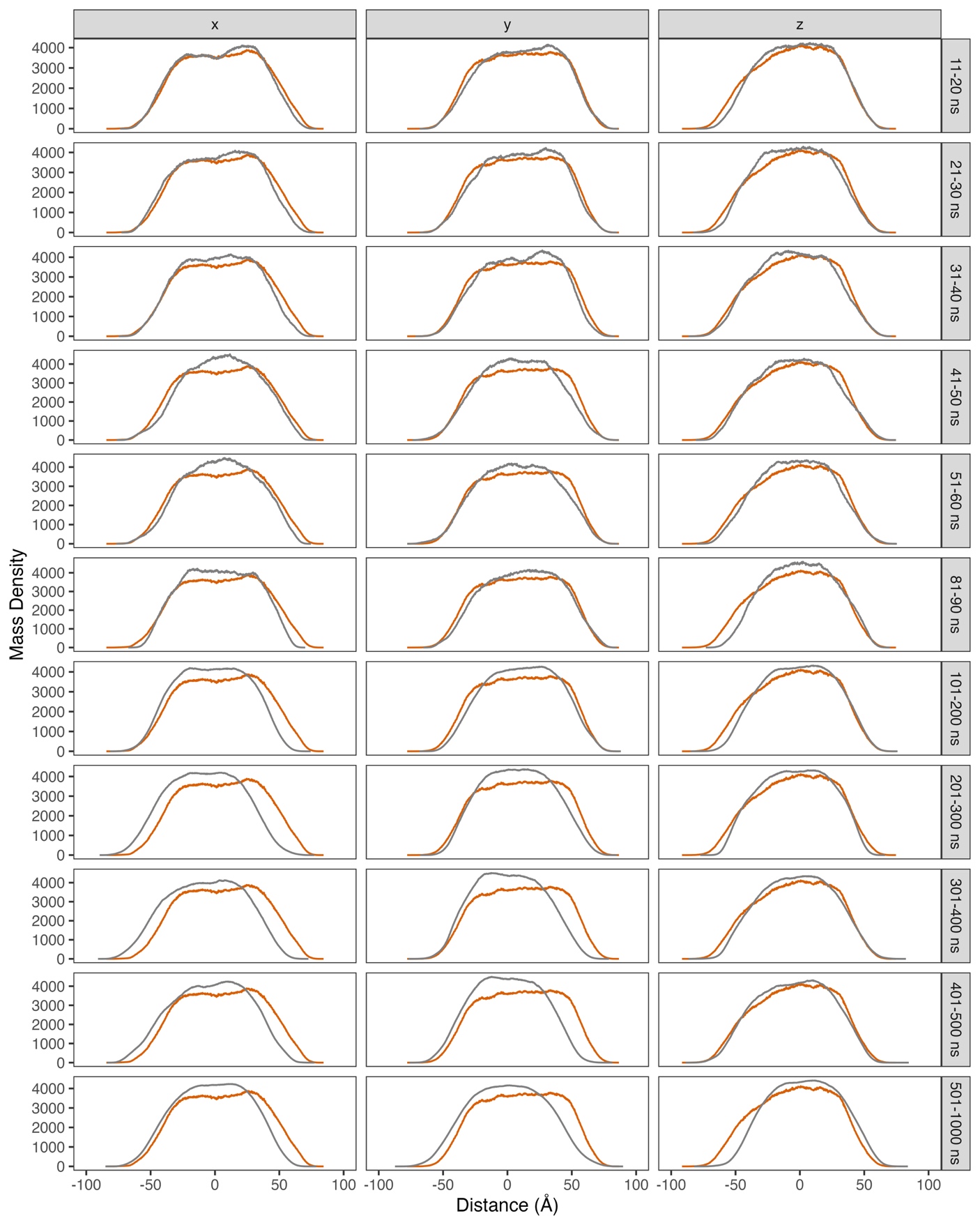


**Figure S2** Mass density profiles of all lipid components of LNP in the SM-102 Positive system in three directions of axes over various time ranges from triplicate 1000-ns trajectories. The orange lines denote the mass density profiles of all lipid components at the time range of 0-10 ns.


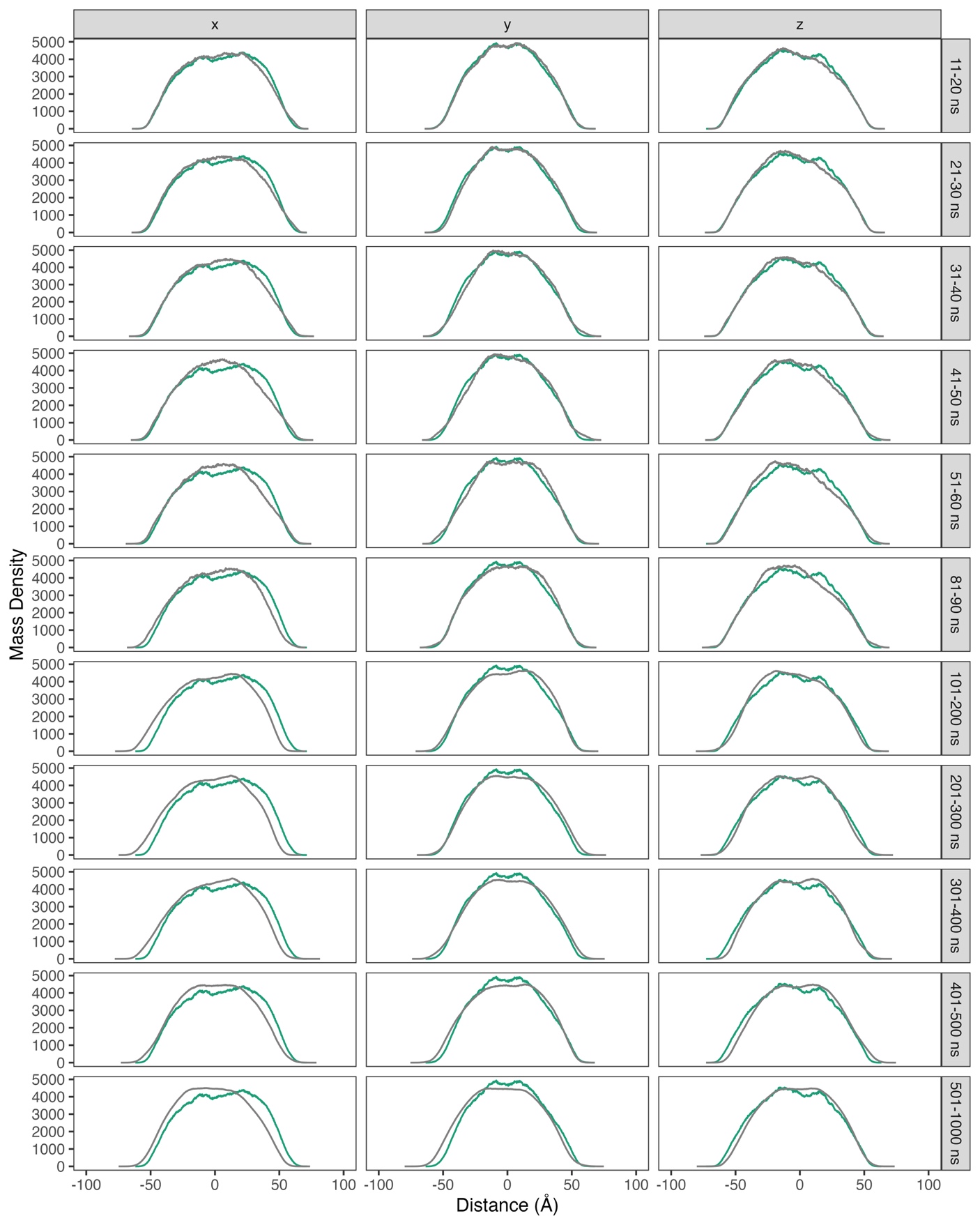


**Figure S3** Mass density profiles of all lipid components of LNP in the SM-102 Neutral system in three directions of axes over various time ranges from triplicate 1000-ns trajectories. The green lines denote the mass density profiles of all lipid components at the time range of 0-10 ns.


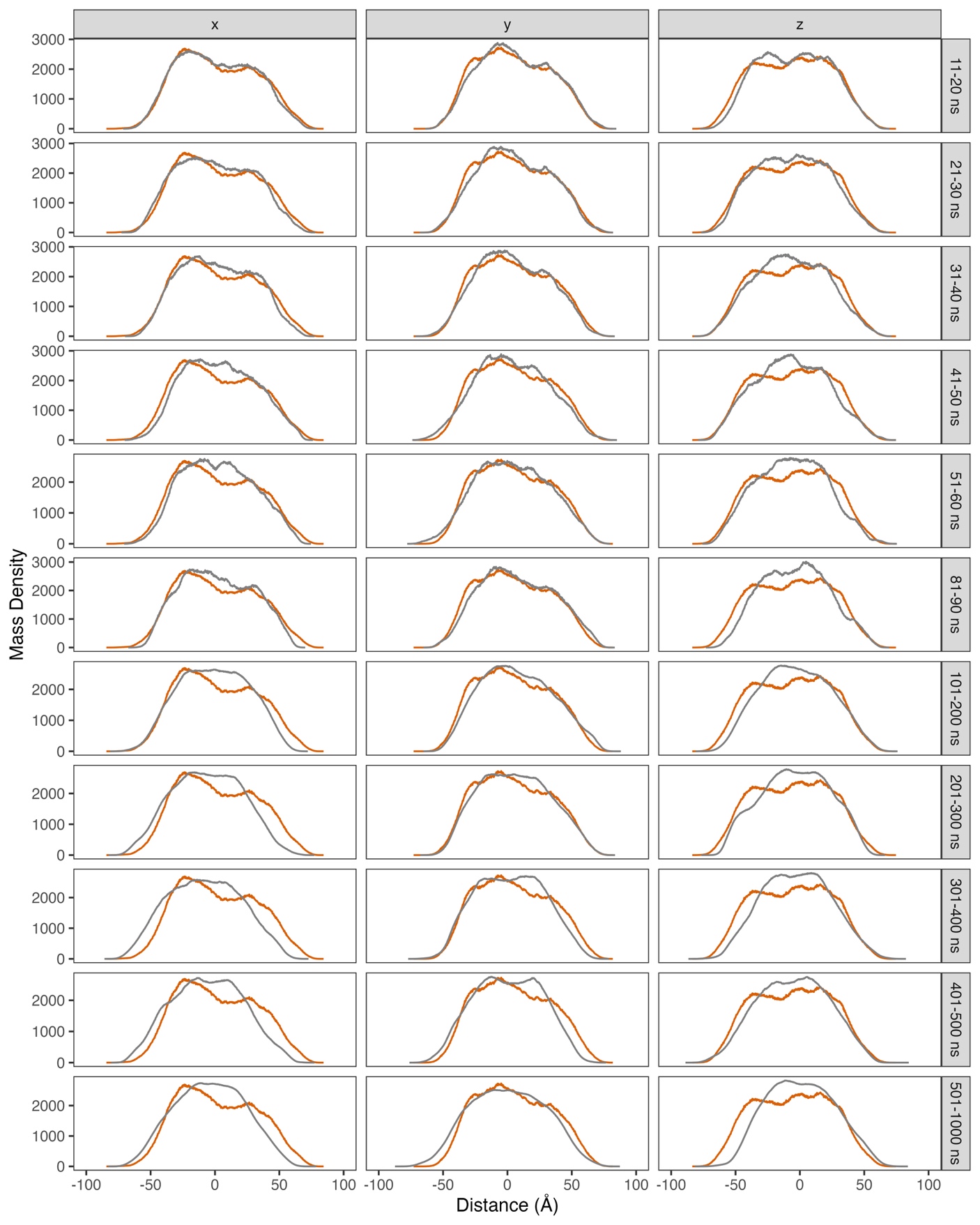


**Figure S4** Mass density profiles of SM-102 lipid of LNP in the Positive system in three directions of axes over various time ranges from triplicate 1000-ns trajectories. The orange lines denote the mass density profiles of SM-102 lipid at the time range of 0-10 ns.


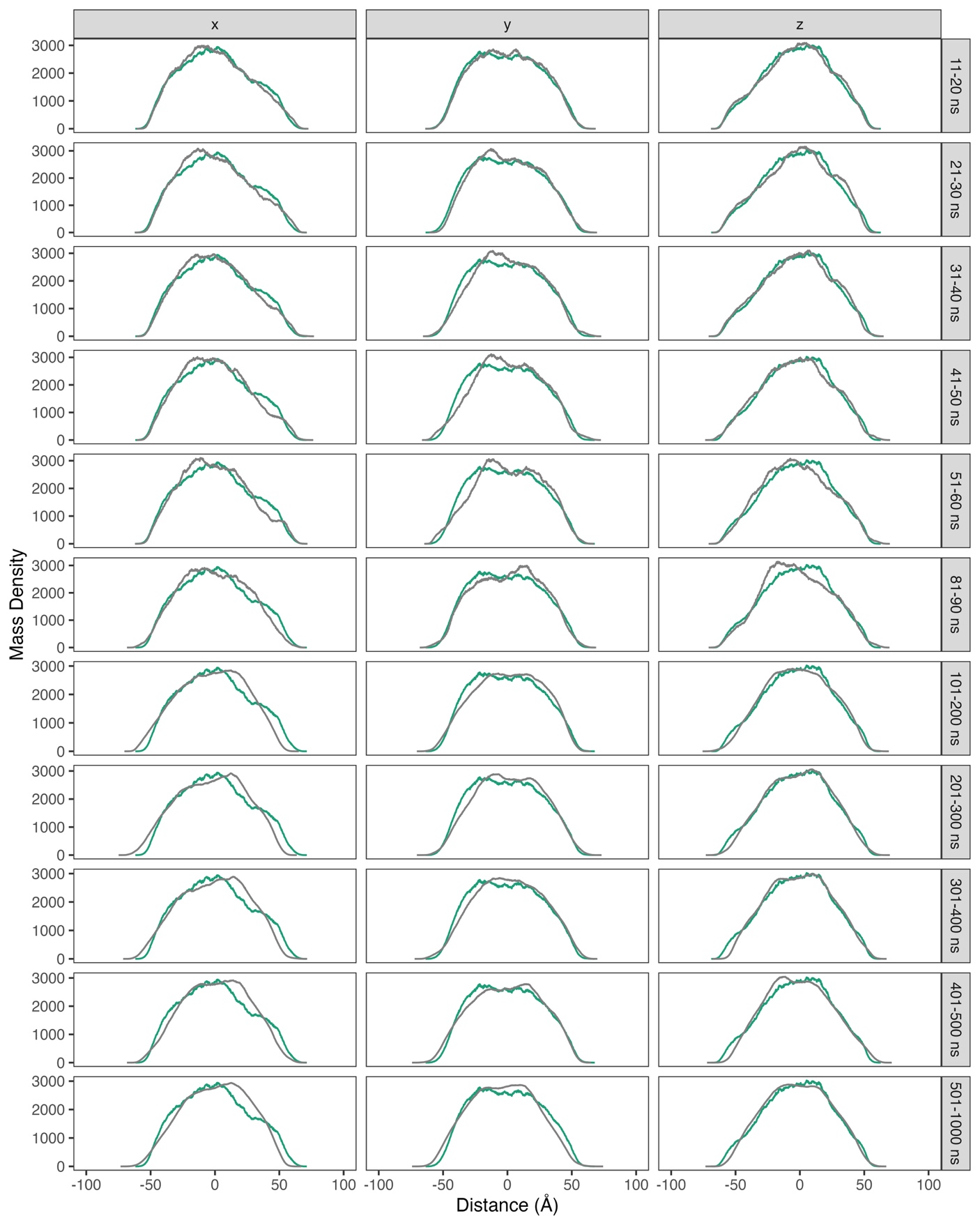


**Figure S5** Mass density profiles of SM-102 lipid of LNP in the SM-102 Neutral system in three directions of axes over various time ranges from triplicate 1000-ns trajectories. The green lines denote the mass density profiles of SM-102 lipid at the time range of 0-10 ns.


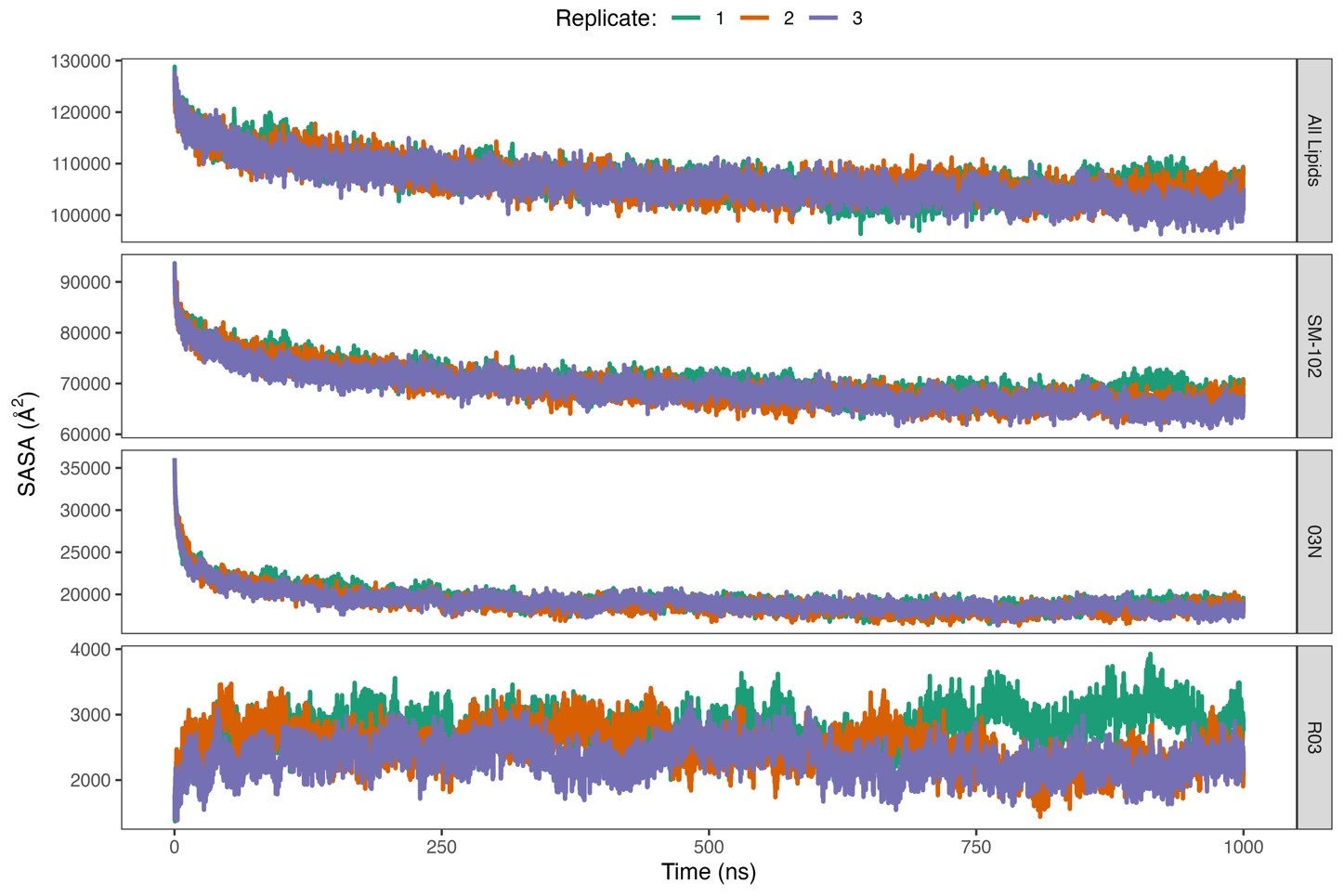


**Figure S6** SASA plots of SM-102 and its charge states in the Neutral system. SM-102P is positively charged SM-102, whereas SM-102N is the neutral charged form. SM-102 is the combined of SM-102P and SM-102N. R03 is the moeity of SM-102P bearing the positive charge, where in SM-102N, its charge is neutral, and it is denoted as 03N. The green, orange, and purple lines represent replicate 1, 2, and 3, respectively.

| 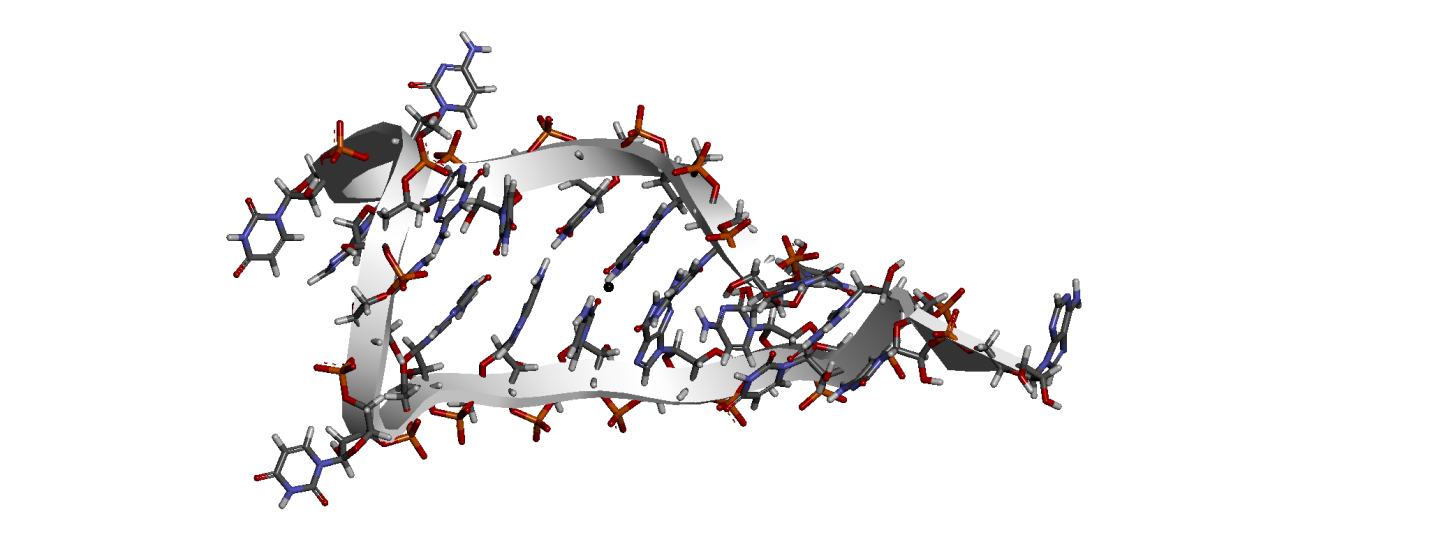 | 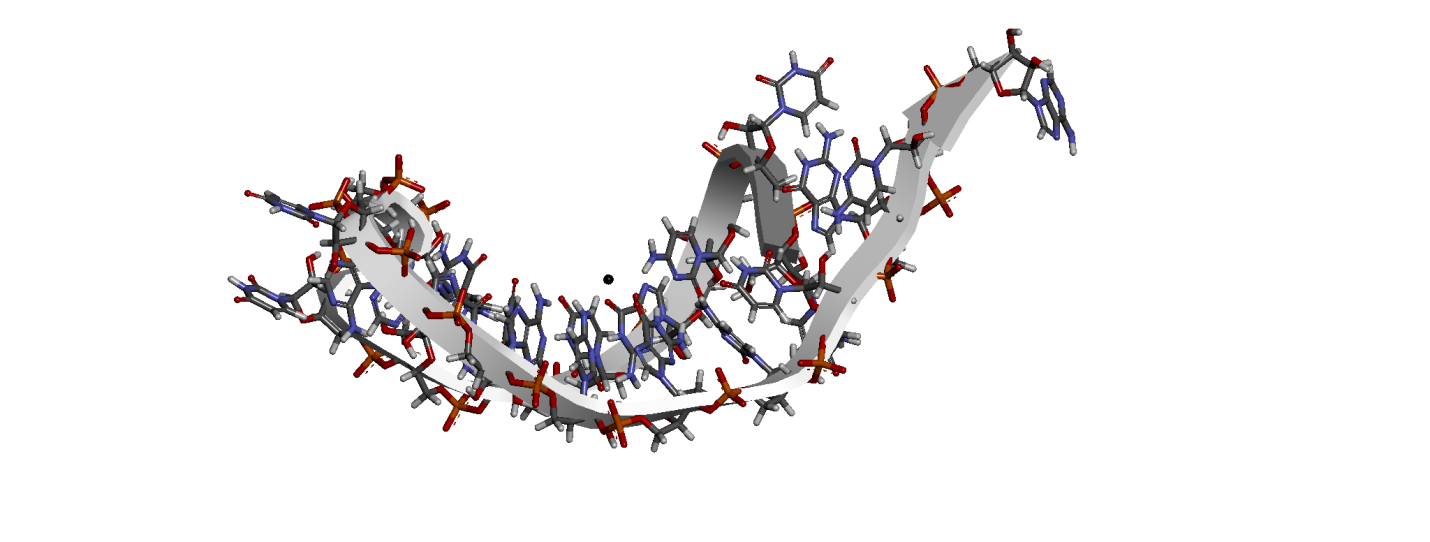 |
| --- | --- |
| 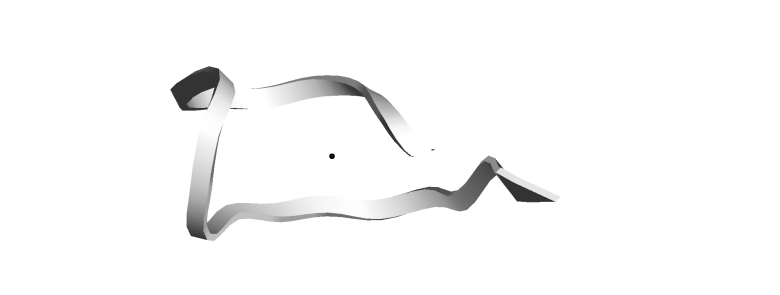 | 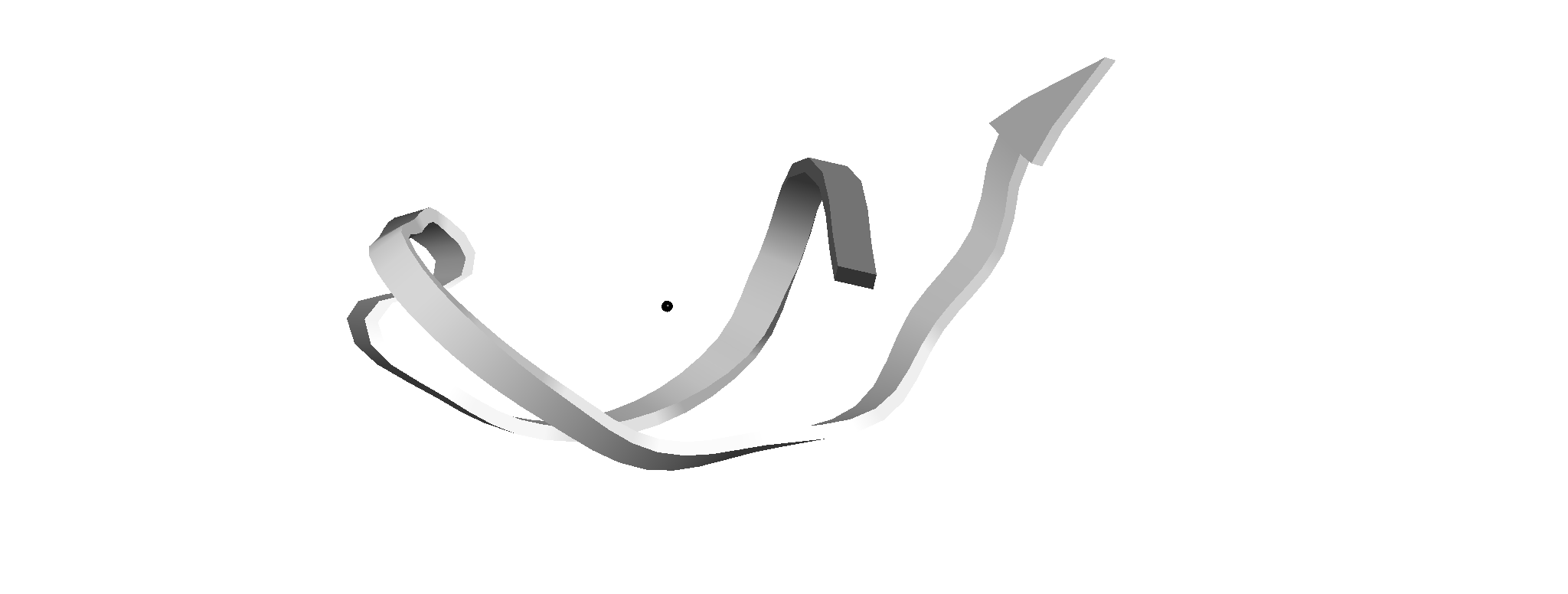 |

**Figure S7** Visualizations of mRNA and its center of mass (black dot).


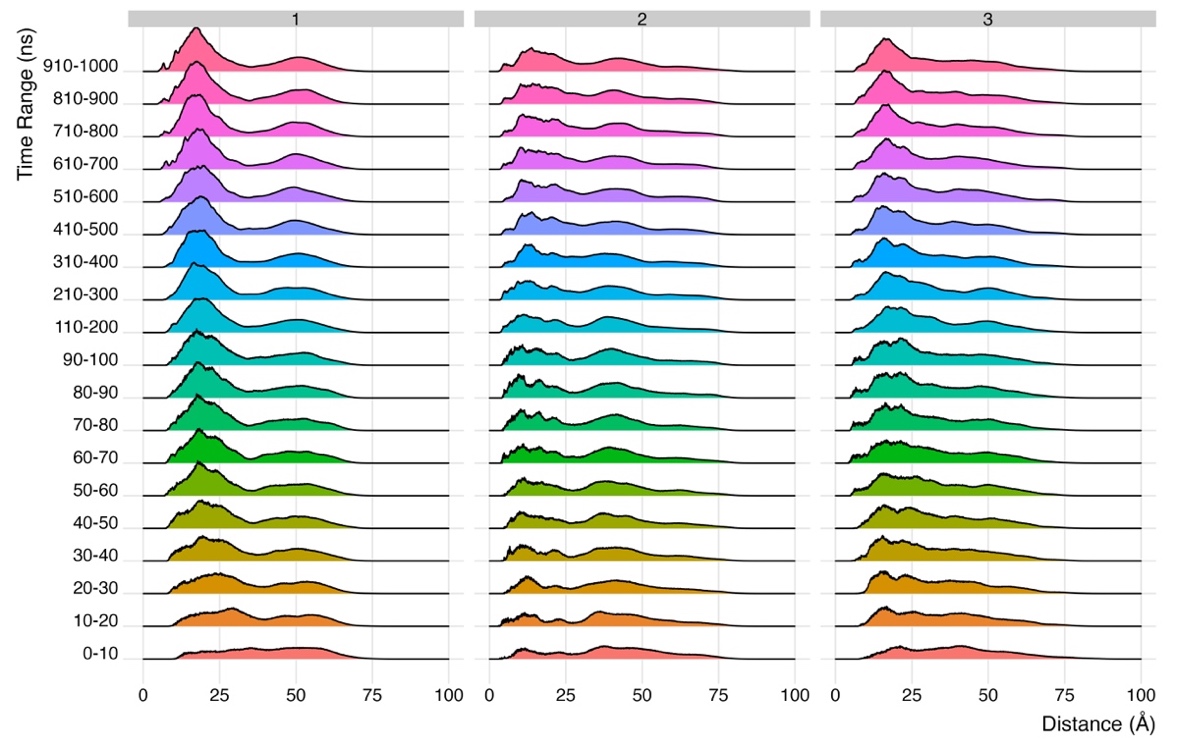


**Figure S8** Radial distribution functions between the center masses of mRNA and R03 in the Positive system over various time ranges of triplicate trajectories. R03 is the part of SM-102 bearing a charge of +1.


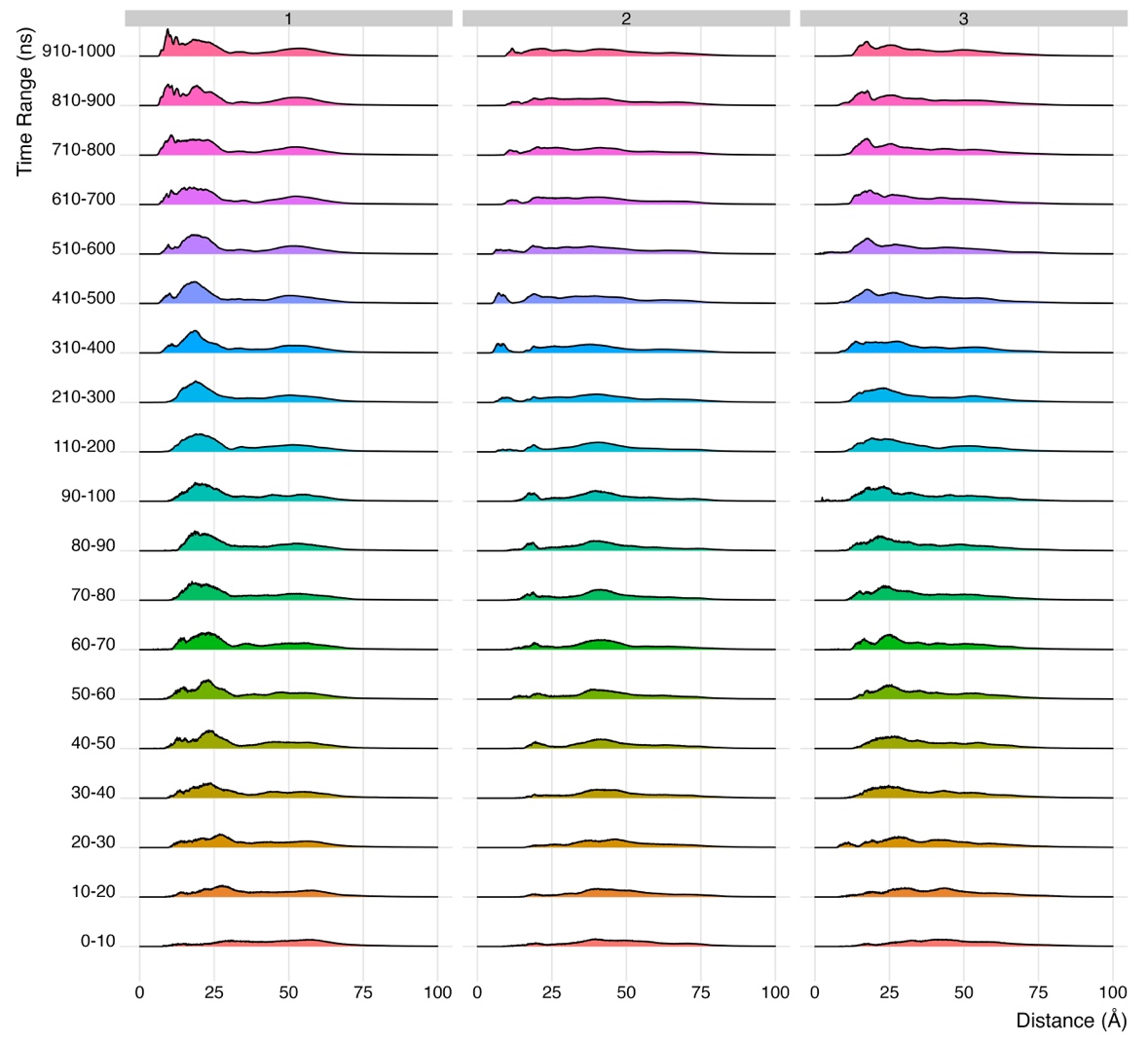


**Figure S9** Radial distribution functions between the center masses of mRNA and citric ions in the Positive system over various time ranges of triplicate trajectories. The citric ions have charges of –1.


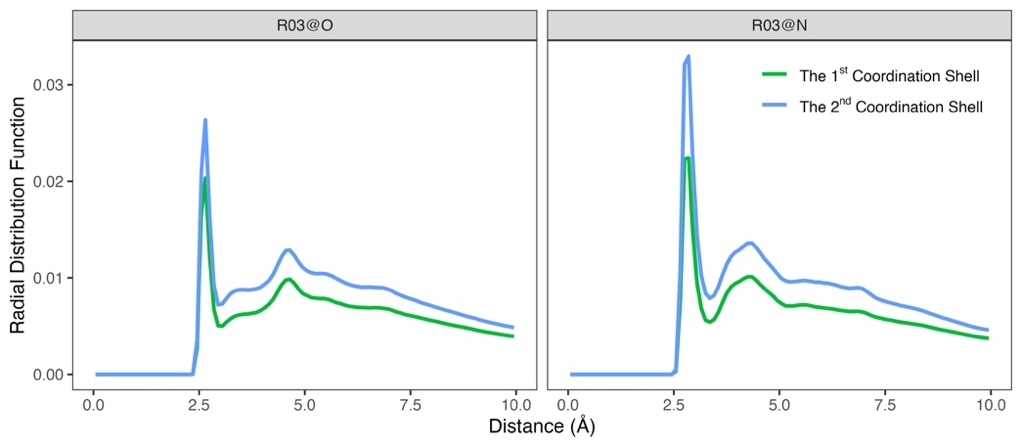


**Figure S10** Radial distribution functions between the R03 headgroup (specifically its oxygen (left) and nitrogen (right) atoms, denoted as O^R03^ and N^R03^) and the oxygen atom of citrate (−1) (O^citrate (−1)^) in the first and second coordination shells in the Positive system. Radial distribution functions were calculated from triplicate trajectories. The green and blue lines represent the first and second coordination shells, respectively.


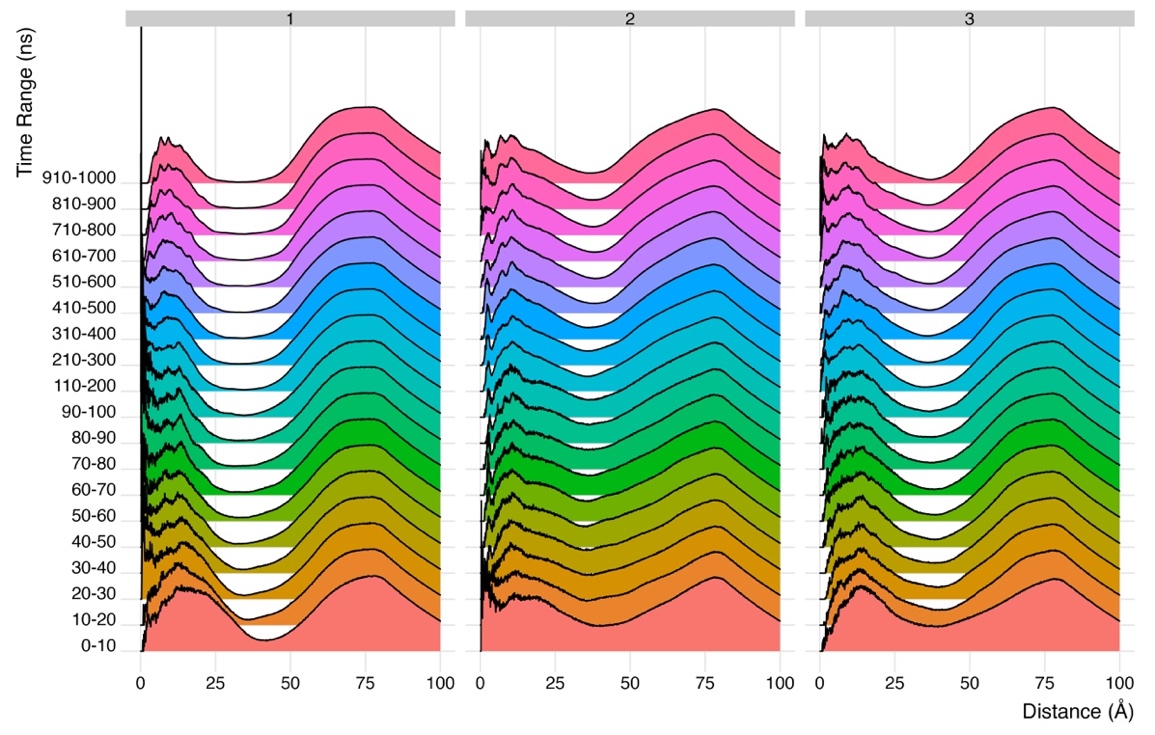


**Figure S11** Radial distribution functions between the center masses of mRNA and water molecules in the SM-102 Positive system over various time ranges of triplicate trajectories.


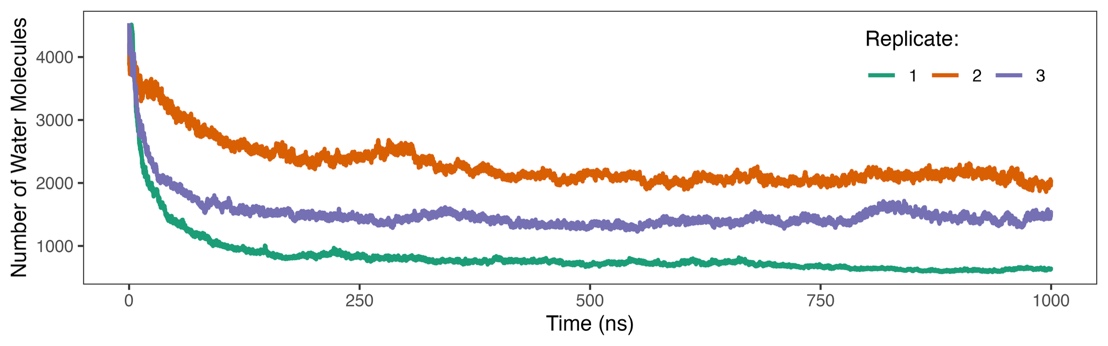


**Figure S12** Number of water molecules around Radial distribution functions between the center masses of mRNA and water molecules in the SM-102 Positive system over various time ranges of triplicate 1000-ns trajectories. The green, orange, and purple lines represent replicate 1, 2, and 3, respectively.


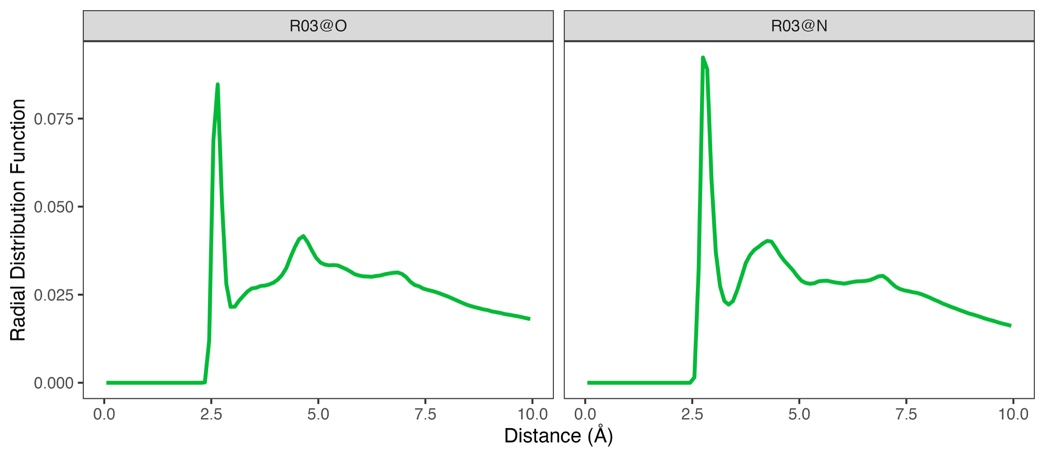


**Figure S13** Radial distribution functions between oxygen (left) and nitrogen (right) atoms in R03 (O^R03^ and N^R03^) and oxygen atoms in citrate (−1) (O^citrate (−1)^) in the first coordination shells in the Neutral system. Radial distribution functions were calculated from triplicate trajectories.


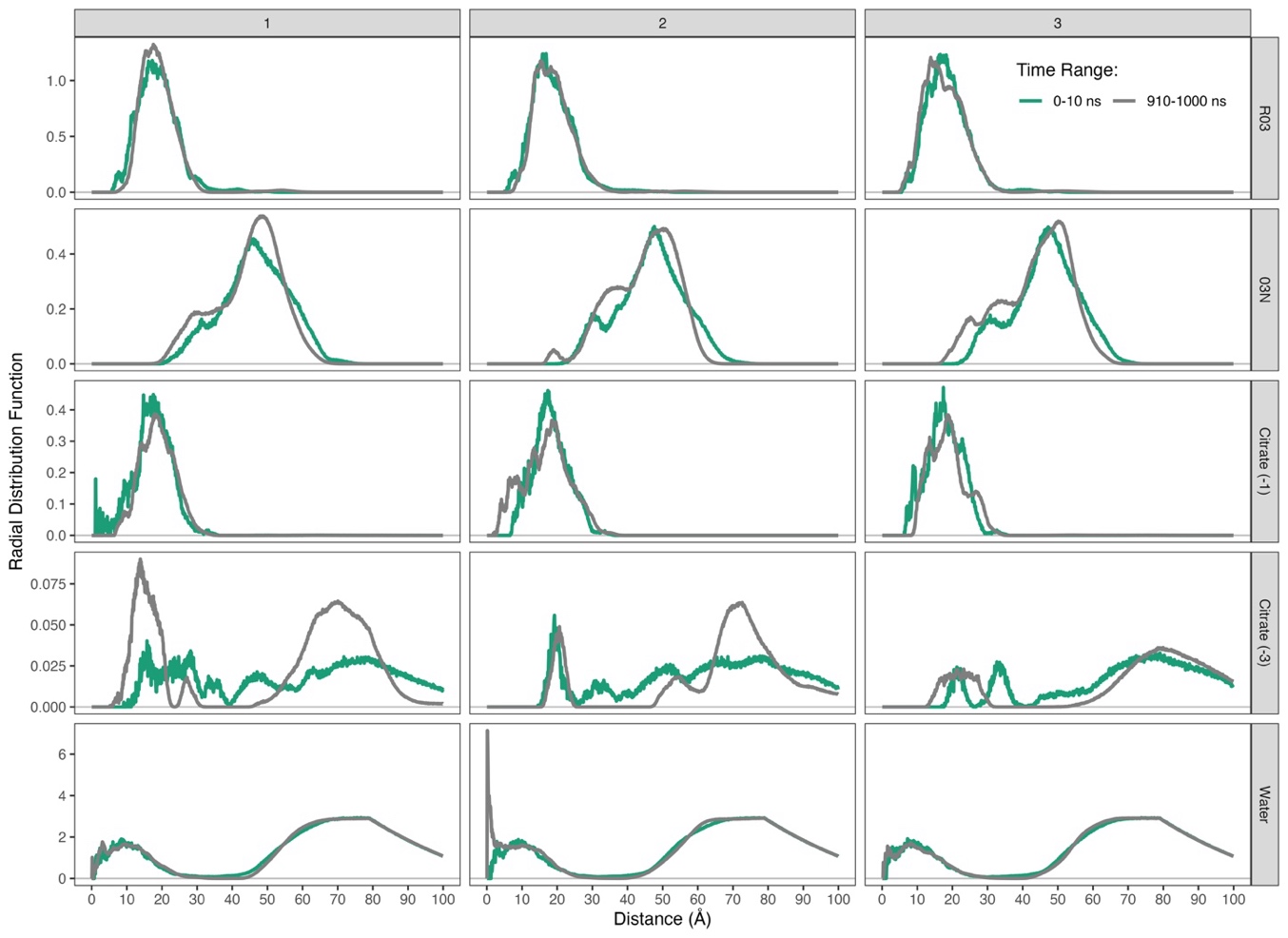


**Figure S14** Radial distribution functions between the center masses of mRNA and some molecules in the Neutral system over various time ranges of triplicate trajectories. The molecules include SM-102 molecules having charges of +1.


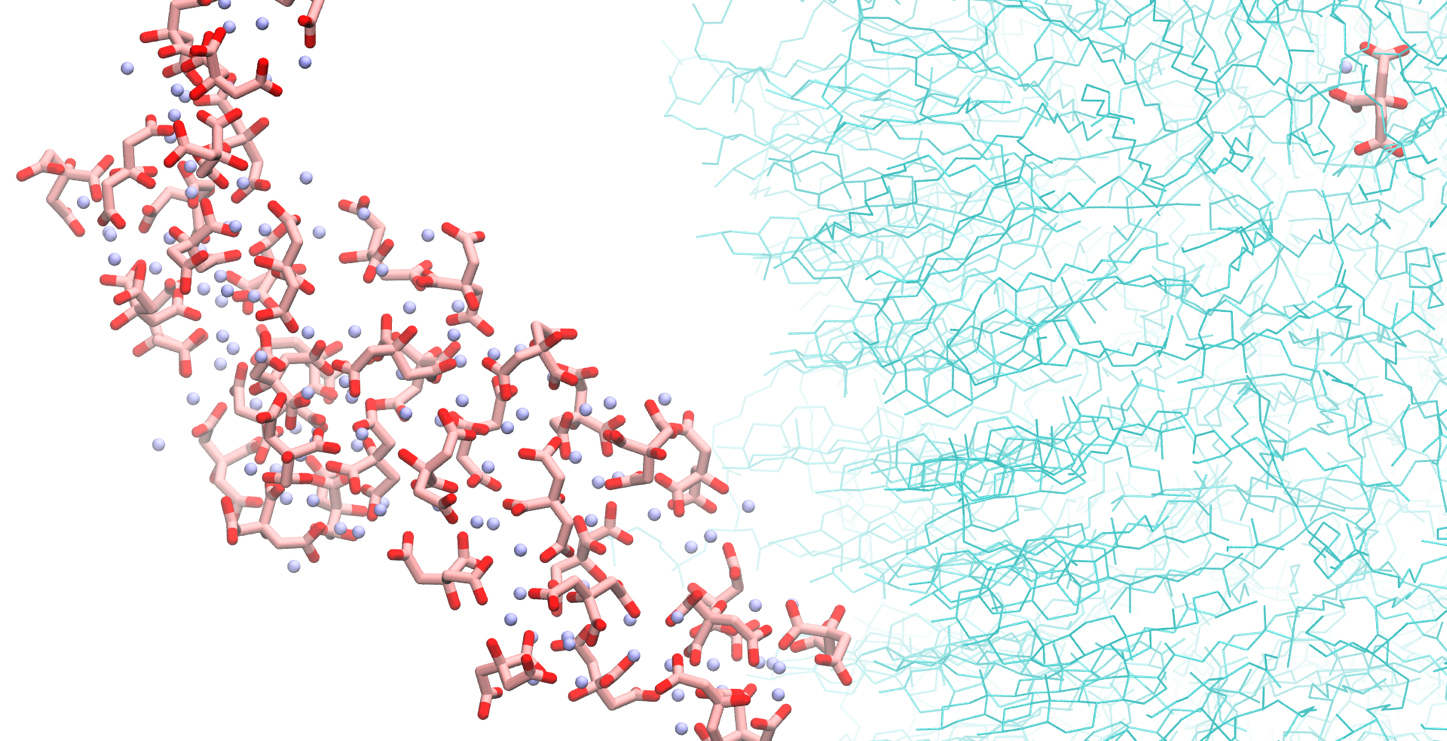


**Figure S15** Coordination formed between citrate (−3) and sodium ions surrounding the outer surface of the LNP. LNP is shown in the cyan line representation, while citrate (−3) ions are in the licorice representation. Sodium ion is blue color, while oxygen and carbon atoms are red and pink colors, respectively.


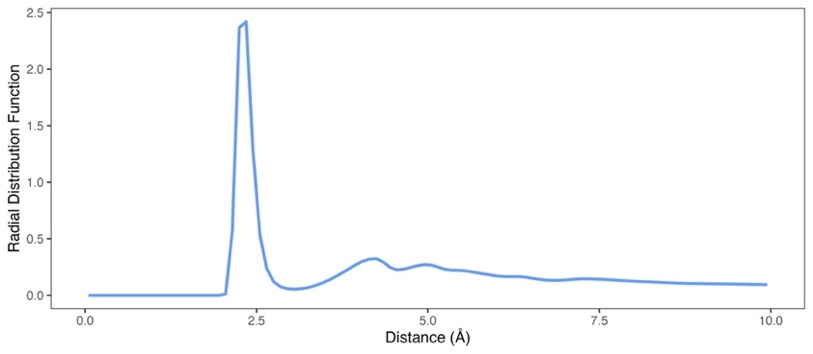


**Figure S16** Radial distribution functions between citrate (−3) and sodium ions surrounding the outer surface of the LNP in the Neutral system over various time ranges of triplicate trajectories.


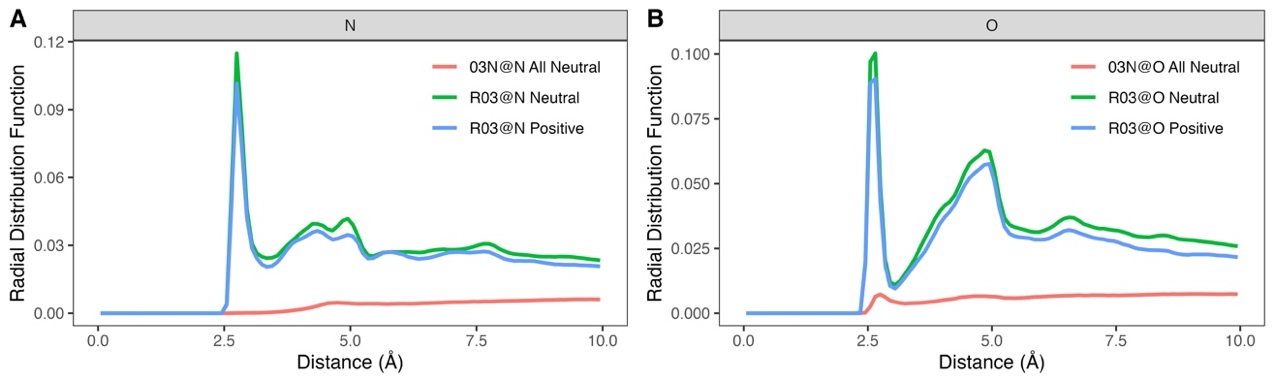


**Figure S17** Radial distribution functions between the R03 headgroup and the oxygen atoms of mRNA (O^mRNA^) in the Neutral, Positive, and All-Neutral systems over various time ranges of triplicate trajectories. Nitrogen (left panel) and oxygen (right panel) atoms were used as the probes for R03 headgroup. Blue and green lines denote the R03 headgroups in the Positive and Neutral systems, respectively. Red lines indicate the R03 headgroup in the neutral charged (03N) in All Neutral system, where R03 headgroups at the inner and outer part of LNP are as 03N.


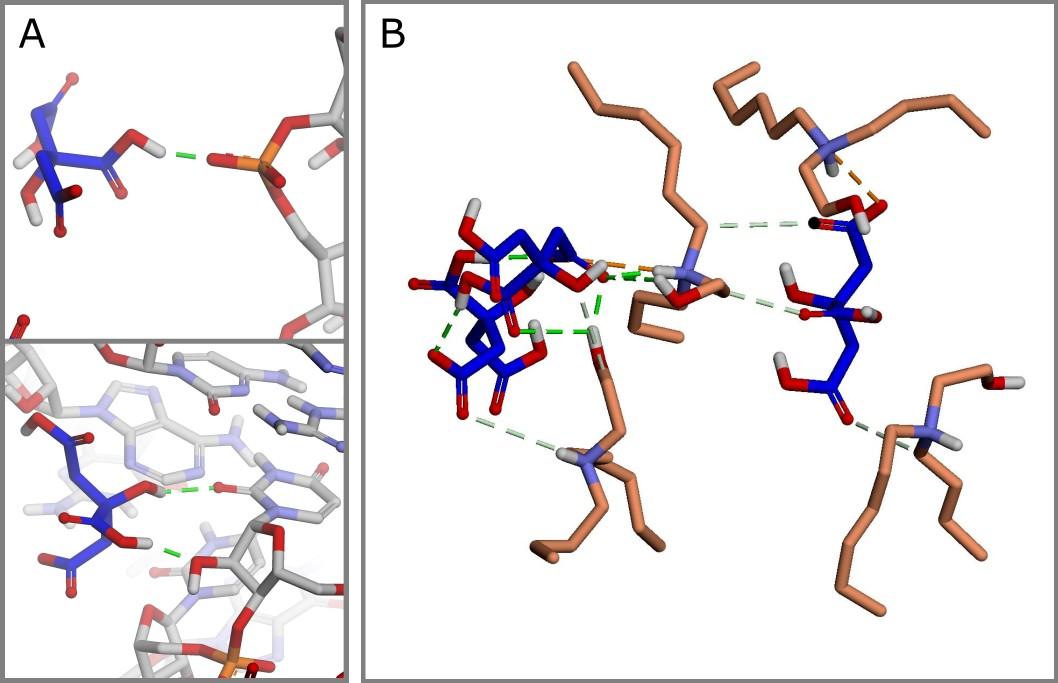


**Figure S18** (A) The interactions between citrate (−1) ions and the mRNA in the Positive system. (B) The interactions between R03 and citrate (−1) ions in the Positive system. Carbon atoms of citrate (−1) ions and the nucleotide residues of mRNA are in blue and grey, respectively, whereas those of R03 are in orange.


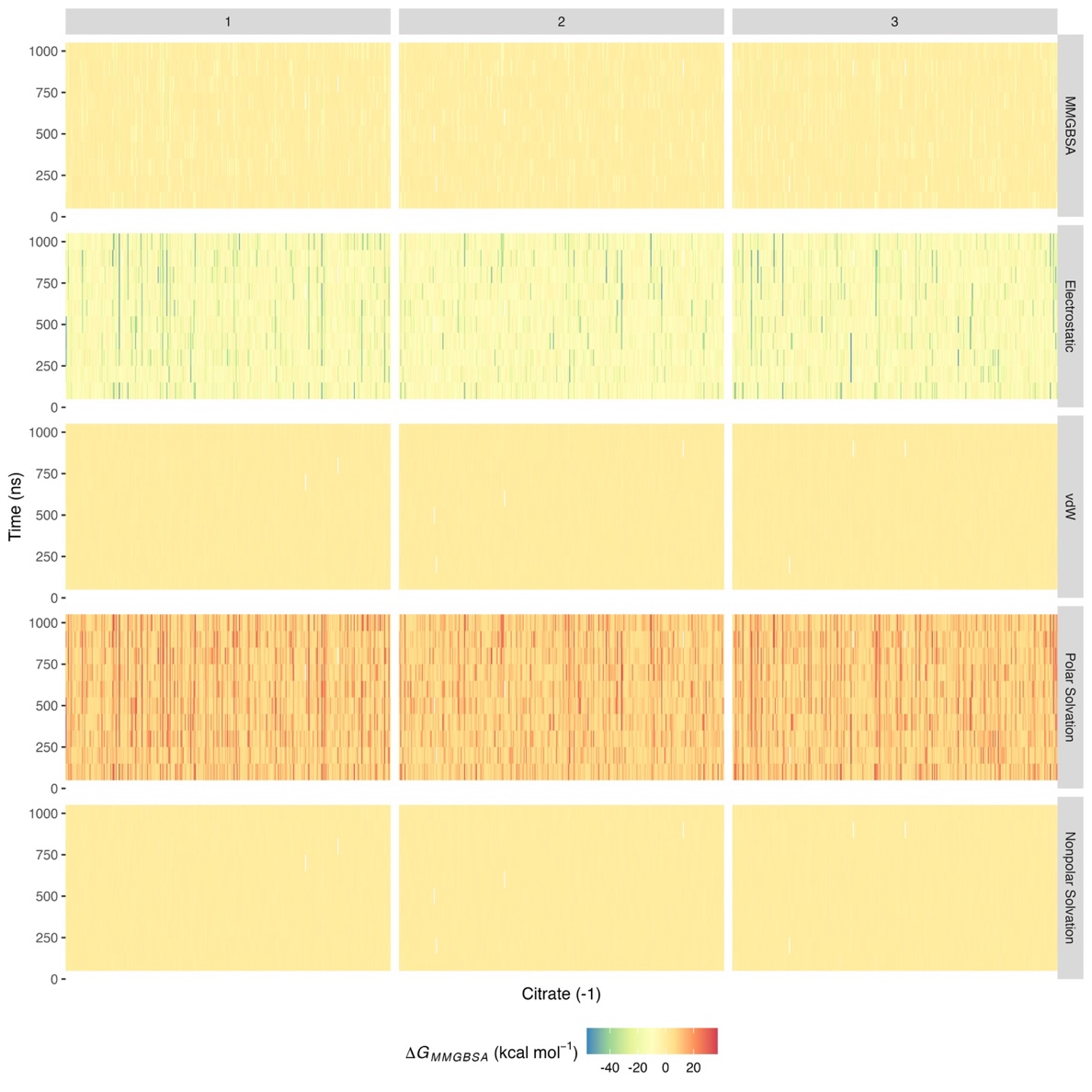


**Figure S19** MMGBSA interaction energy terms of R03 with citrate (–1) ions in the Positive system from calculated from triplicates 1000-ns trajectories.


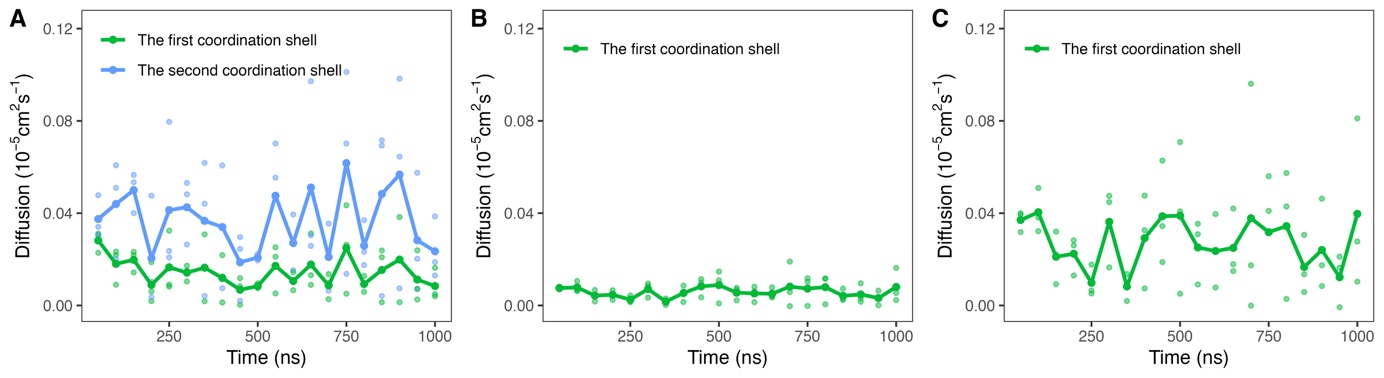


**Figure S20** Diffusion constants of SM-102 in the Positive and Neutral systems, where R03 and 03N act as the probes, at 50-ns intervals over 1000 ns. (A) The diffusion constants of R03 in the Positive system. Coordination shell 1 refers to the RDF curve at 2.85-33.75 Å, whereas coordination shell 2 is that at 33.75-88.25 Å (see Figure 2C). (B) The diffusion constants of R03 in Neutral system. (C) The diffusion constants of 03N in the Neutral system. Diffusion constants were calculated from triplicate trajectories. Lines denote diffusion constant average values, while dots are diffusion constants from triplicate trajectories. Green and blue represent the first and second coordination shell, respectively.


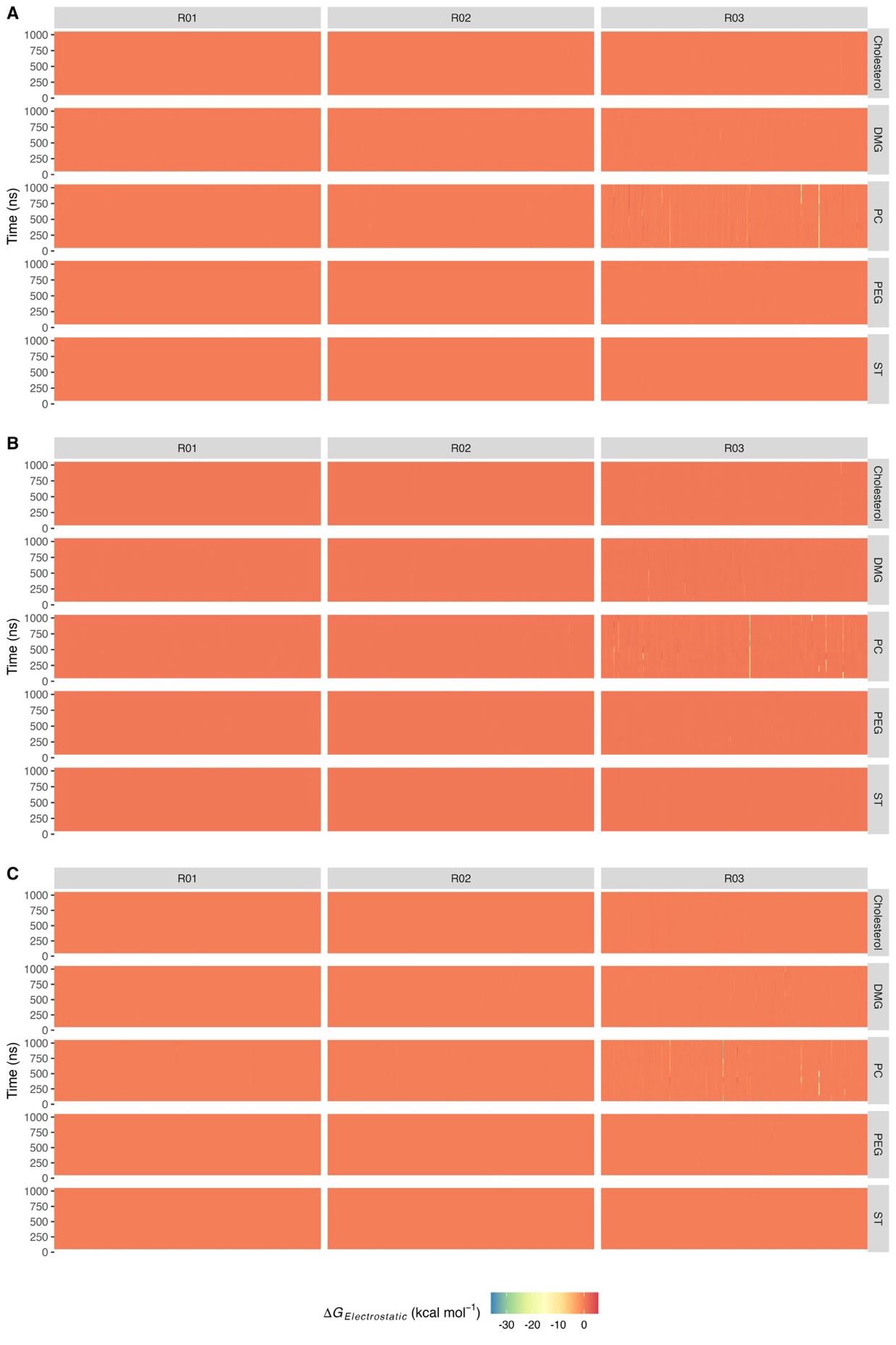


**Figure S21** The electrostatic interaction energy of R01, R02, and R03 with lipids in the Positive systems from the first (A), second (B), and third (C) replicate trajectories.


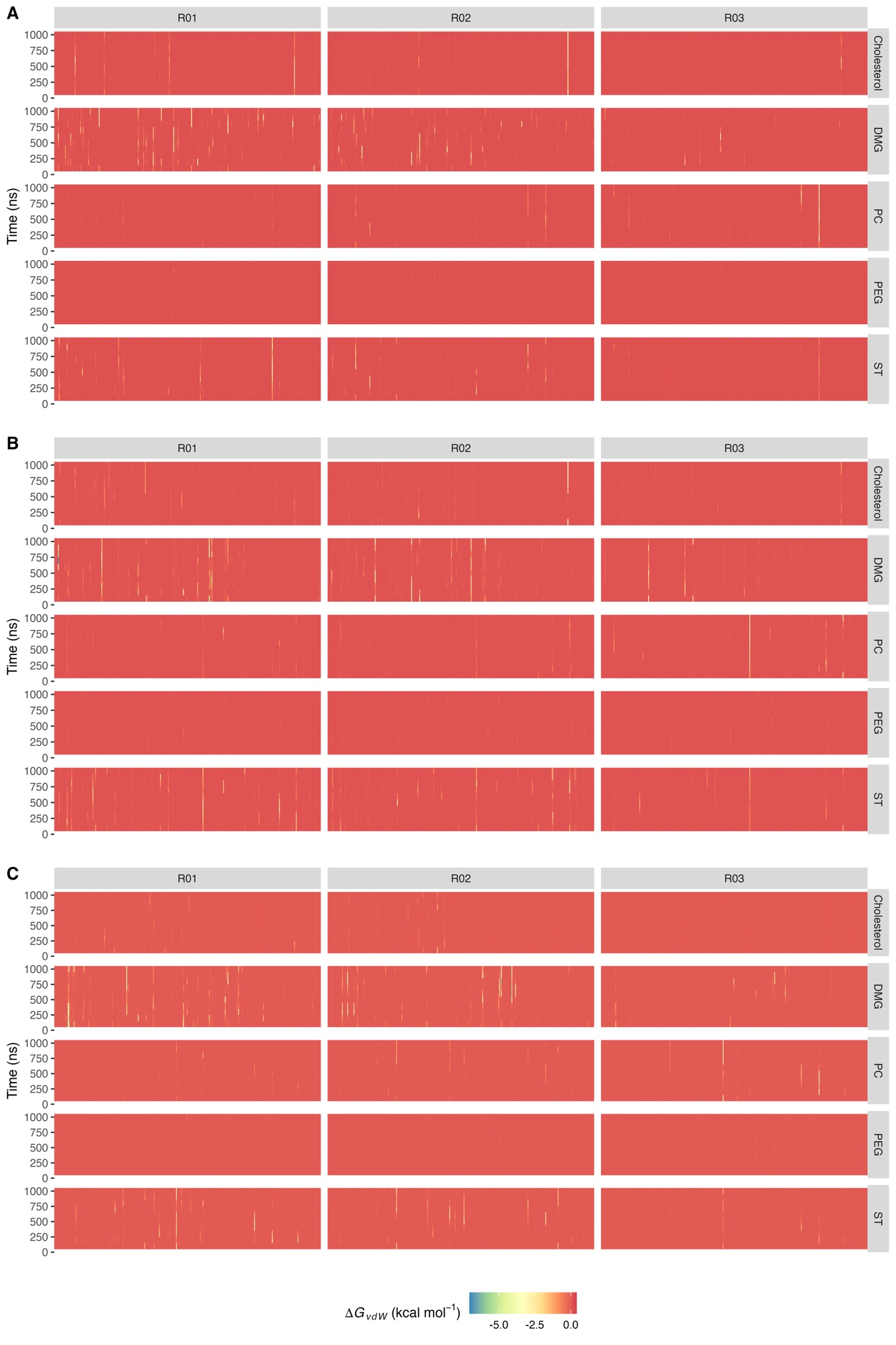


**Figure S22** The van der Waals interaction energy of R01, R02, and R03 with lipids in the Positive systems from the first (A), second (B), and third (C) replicate trajectories.


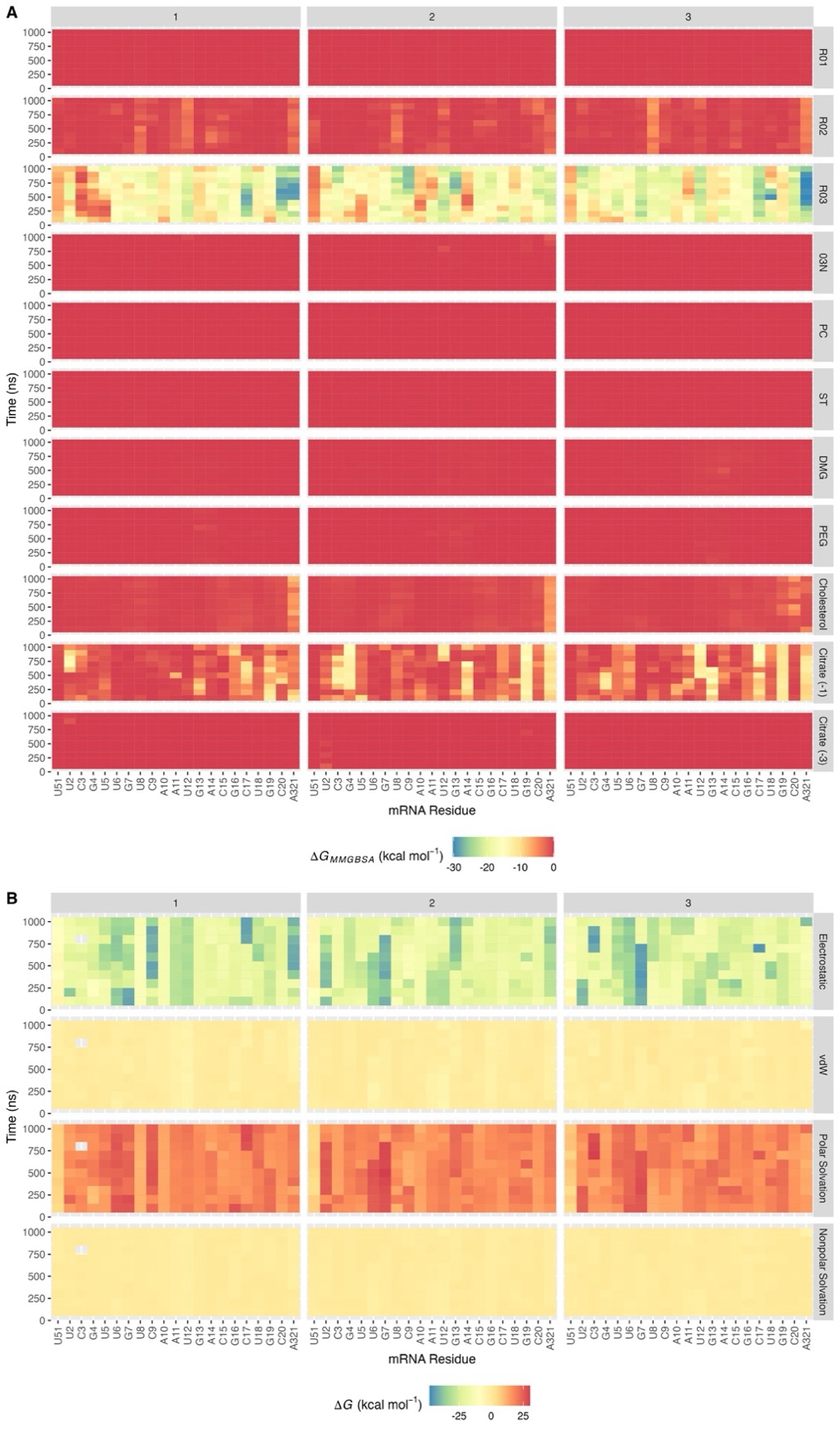


**Figure S23** MMGBSA energy decomposition analysis (EDA) and interaction energy terms between mRNA and other components in the Neutral systems from triplicate 1000-ns trajectories. (A) EDA between mRNA residues and other components. R01 and R02 are hydrophobic chains of SM-102 (see Figure S1); R03 is the part of SM-102P bearing a positive charge, while 03N is the neutral form of R03 in SM-102N; ST and PC refer to stearoyl (18:0) and phosphatidylcholine, respectively, which form DSPC; DMG is dimyristoyl (14:0) glycerol, while PEG is polyethylene glycol, which are parts of DMG-PEG2000 (see Figure S1); Citrate (−1) and (−3) is the citrate ions with charges of −1 and −3, respectively. (B) MMGBSA interaction energy terms between R03 and each mRNA residue. The energy values were computed at 100-ns intervals across 1000-ns trajectories. Numerical labels above each column in the graph denote replicates 1, 2, and 3.

**
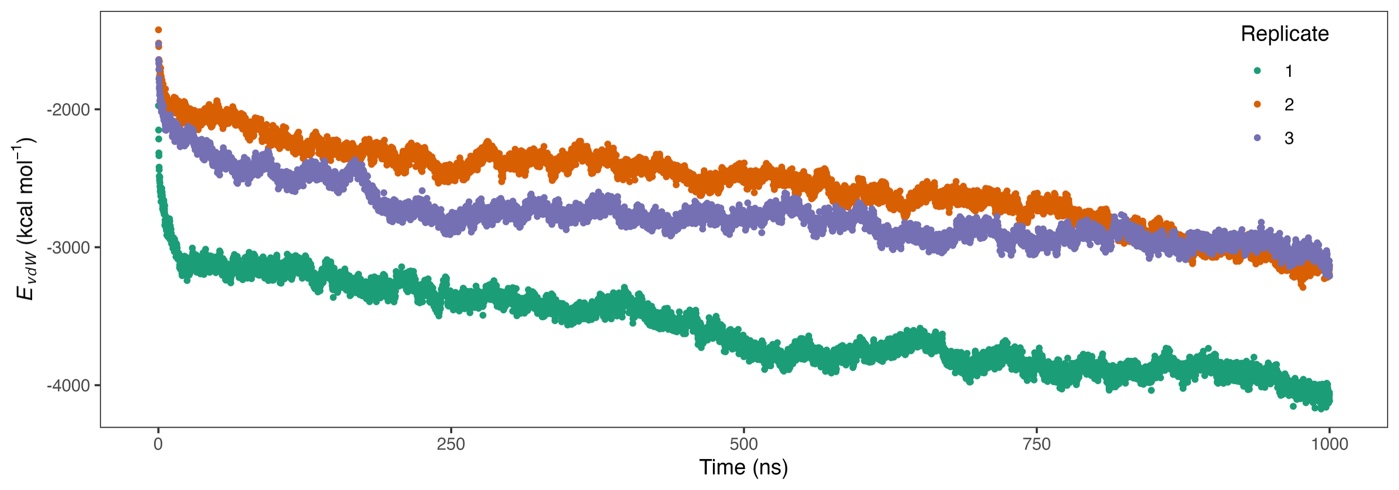
**

**Figure S24** LIE of van der Waals (vdW) energy term for R03 with other lipid components in the first coordination shell of the Positive system. The green, orange, and purple points represent replicate 1, 2, and 3, respectively.
